# Inclusion of Dominance Effects in the Multivariate GBLUP Model

**DOI:** 10.1371/journal.pone.0152045

**Published:** 2016-04-13

**Authors:** Jhonathan Pedroso Rigal dos Santos, Renato Coelho de Castro Vasconcellos, Luiz Paulo Miranda Pires, Marcio Balestre, Renzo Garcia Von Pinho

**Affiliations:** 1 Department of Biology (DBI), Federal University of Lavras, Lavras, Minas Gerais, Brazil; 2 Department of Exact Science (DEX), Federal University of Lavras, Lavras, Minas Gerais, Brazil; 3 Department of Agriculture (DAG), Federal University of Lavras, Lavras, Minas Gerais, Brazil; China Agricultrual University, CHINA

## Abstract

New proposals for models and applications of prediction processes with data on molecular markers may help reduce the financial costs of and identify superior genotypes in maize breeding programs. Studies evaluating Genomic Best Linear Unbiased Prediction (GBLUP) models including dominance effects have not been performed in the univariate and multivariate context in the data analysis of this crop. A single cross hybrid construction procedure was performed in this study using phenotypic data and actual molecular markers of 4,091 maize lines from the public database Panzea. A total of 400 simple hybrids resulting from this process were analyzed using the univariate and multivariate GBLUP model considering only additive effects additive plus dominance effects. Historic heritability scenarios of five traits and other genetic architecture settings were used to compare models, evaluating the predictive ability and estimation of variance components. Marginal differences were detected between the multivariate and univariate models. The main explanation for the small discrepancy between models is the low- to moderate-magnitude correlations between the traits studied and moderate heritabilities. These conditions do not favor the advantages of multivariate analysis. The inclusion of dominance effects in the models was an efficient strategy to improve the predictive ability and estimation quality of variance components.

## Introduction

Several factors can complicate the process of selection and recommendation of cultivars in a maize breeding program. One of the most severe factors is the potentially large number of hybrids that can be obtained from a relatively small set of lines. This number of crosses complicates the choice of the formation of combination pairs, even when well-established heterotic groups are available. Should the breeder perform all or most of these combinations, the high financial costs and operational difficulties under testing conditions with several replicates and installed at a high number of sites would render the evaluation of these hybrids financially unfeasible. For example, the combination between 100 lines of one heterotic group and 100 lines of another heterotic group allows 10,000 combinations of simple hybrids that must be evaluated at several sites and with replicates. Accordingly, the availability of tailor-made methods of prediction of the best combinations between candidate lines may be a key point in maximizing the use of financial resources in breeding programs.

In recent years, the development of large-scale genotyping methods—such as Genotyping-by-sequencing (GBS), Dart Array Technology (DArT) and Restriction site Associated DNA (RAD) markers [1–3]—the steady decrease in costs per data-point and the strong trend toward increased spending on phenotyping has encouraged a growing interest in breeding programs to use prediction processes based on data from molecular markers [4–6].

The progress of these molecular methods occurred concurrently with the development of statistical and computational methods capable of handling the large amount of data generated by these markers with genome-wide coverage. New regression models have recently been proposed for the analysis of phenotypic data and molecular markers in breeding programs. These models are integrally applied to selection and prediction in a process commonly known as genomic selection (GS) [7]. The main premise of this method is to cover the genome extensively using molecular markers and to seek relationships between markers and phenotypic variants of a population, regardless of whether those markers belong to causal genomic regions [8,9]. This method is advantageous from a practical standpoint because it does not require the adoption of constructions and specific population structures, unlike traditional methods that use markers, such as the Quantitative Trait Loci (QTL) mapping process [10].

Multiple regression models, mixed models, ridge regression, semi-parametric methods and Bayesian methods are generally used frequently in GS [11]. After several studies, few differences were observed among these methods in the estimation of additive genetic effects, with marginal differences depending on the species and the genetic architecture of the trait evaluated [12–15]. Bayesian methods enable the estimation of genetic variances specific to the genetic effects of markers and the inclusion of a priori data on the probability distributions of genetic effects. Conversely, mixed models may be used by direct regression of the markers by applying a method called ridge regression BLUP (rrBLUP) or by using the Genomic BLUP (GBLUP) method. The latter method recovers genetic data between individuals by kinship matrices designed using molecular markers. These matrices designed using markers have been found to be more advantageous than matrices estimated using pedigrees, primarily because they recover data based on both Mendelian sampling and close relatives, enabling the use of genetic relationships from the most recent generations to the most distant [6].

In these models, GS is usually applied directly to the target trait; however, direct genomic selection may be ineffective depending on the heritability and complexity of the genetic architecture [8,16]. Accordingly, indirect genomic selection may be used to improve the accuracy of prediction using simpler heritability traits and with high correlation with the target trait. Several studies report improved predictive ability using models analyzing data on high-heritability secondary traits together with the target trait [17–19]. These models are known as multi- or multivariate traits. There are virtually no reports on the use of this class of model in studies using annual crop species [19]. Reports on the use of multivariate models that analyze kinship relationships estimated using molecular markers are even less common [20–22]. In the context of GS, the multivariate GBLUP (GBLUP-MV) model [17] enables the use of data between secondary traits by means of the covariance between those traits and between individuals [21,22]. There is a lack of studies evaluating the usefulness of these models, especially in the case of maize.

In addition to indirect selection, which may be included in multi-trait models, the inclusion of dominance effects in the model to take advantage of the heterosis phenomenon is another factor that may be used by breeders to increase predictive ability. In the context of the GBLUP model, the inclusion of dominance effects may be easily performed using the kinship matrix including dominance estimated using molecular markers without drastically changing the model size, as would occur in a regression model using markers [18].

The proposal of a model using dominance data and multiple traits in the context of GS has not been reported in the literature thus far. Accordingly, this study aims to (i) construct single cross maize hybrids using actual phenotypic and genetic data based on maize lines while preserving the genetic architecture of the traits; (ii) propose the inclusion of dominance effects in the multivariate GBLUP (GBLUP-MV-AD) model; and (iii) compare the GBLUP-MV-AD model with the multivariate additive GBLUP (GBLUP-MV-A), univariate additive GBLUP (GBLUP-UV-A), and univariate GBLUP models with the inclusion of dominance effects (GBLUP-UV-AD) regarding the predictive ability and estimation of genetic parameters.

## Materials and Methods

### Panzea database of maize lines

This study used single-nucleotide polymorphisms/genotyping by sequencing (SNPs/GBS) data and phenotyping of lines derived from the Nested Association Mapping population (NAM) of the germplasm bank of the United States Department of Agriculture-Agricultural Research Service (USDA-ARS), genetically characterized by [23]. This is an open-access database, available online through the platform Panzea (http://www.panzea.org/). The genotypes were obtained from http://www.panzea.org/#!genotypes/cctl using the file ZeaGBSv1.0 and the NAN phenotypes were obtained from http://www.panzea.org/#!phenotypes/c1m50. The NAM population was prepared from crosses between the line B73 and 25 other inbred lines of high genetic diversity. Those crosses were planned to represent the gene pool of those lines.

The number of markers used in this study consisted of a sample of the total number of markers available for that population. Systematic sampling of 27,000 markers uniformly distributed throughout the 10 maize linkage groups was performed in a total group of 681,257 markers. The selection of markers was performed by draw of the first mark in the range between the 1^st^ and 25^th^ marks, and the others were selected linearly every 25 marks until fully covering all linkage groups (2,700 marks selected per chromosome). Lost marks were allocated using the *A*.*mat* function, *mean* method of the rrBLUP package [24] of the R software [25] (R core team, 2014).

Five traits were studied: (i) plant height—PH (4,065 lines); (ii) ear height—EH (4,061 lines); (iii) ear length—EL (3,178 lines); (iv) ear row number—ERN (3,084 lines); and (v) kernel weight—KW (3,313 lines). Phenotypic data were collected in assays conducted in 2006 during the summer harvest in the city of Aurora, New York, at 42.75° N latitude and 76.70° W longitude. The data handling process of the Panzea project was performed using the R software [25] and the Trait Analysis by Association, Evolution and Linkage (TASSEL) software [26].

### Genetic analysis of lines and identification of Quantitative Trait Loci (QTLs)

The Bayes B (BB) method was used to estimate the genetic effects of the 27,000 molecular markers, and individual analyses were performed for each of the five traits of previously reported lines. The genetic model used for BB analysis is shown below:

$$y=j\mu \text{+}W_{\text{A}}a\text{ +}W_{\text{D}}d+e$$ (1)

where **y** is a vector of *n* × 1 observations, and *n* is the number of observations; ***j*** is a fixed-effects (*n*−1) incidence vector, *μ* is the sample mean; **W**_**A**_ and **W**_**D**_ are incidence *n* × *q* matrices of allele substitution **a** (*q* ×*1*) and dominance deviation **d** (*q* ×*1*) effects, wherein *q* is the number of allelic substitutions or dominance deviation effects; and e is the vector of *n* × 1 residual effects. The incidence matrices **W**_*A*_ and **W**_*D*_ were designed following Cockerham’s model [27,28]:

$$W_{\text{A}}\{\begin{array}{c} 2-2p_{k}\\ 1-2p_{k}\\ 0-2p_{k} \end{array}\text{ for the genotypes }\{\begin{array}{c} {\text{A}}^{1}{\text{A}}^{1}\\ {\text{A}}^{1}{\text{A}}^{2}\\ {\text{A}}^{2}{\text{A}}^{2} \end{array}$$

$$W_{\text{D}}\{\begin{array}{c} -2{\left(1-p_{k}\right)}^{2}\\ 2\text{p}(1-p_{k})\\ -2p_{k}{}^{2} \end{array}\text{ for the genotypes }\{\begin{array}{c} {\text{A}}^{1}{\text{A}}^{1}\\ {\text{A}}^{1}{\text{A}}^{2}\\ {\text{A}}^{2}{\text{A}}^{2} \end{array}$$

wherein p_k_ is the frequency of the favorable allele at locus *k*.

A full description of the specifications of the probability distributions of the random effects and Bayes B model parameters may be found in [29] and [8]. All Bayesian analyses were performed in the Bayesian Generalized Linear Regression (BGLR) package [29] of the R software using the *BGLR* function fitted to 10,000 iterations, with the first 1,000 cycles discarded as burn in, as reported in [7]. The mixing parameter π (100/27,000) of the Bayes B model was introduced to fit the 100 allele-substitution effects and 100 dominance effects of marks. All other settings of the BGLR function were maintained in the default mode of the package.

### Building the genomic architecture

The one hundred largest allelic substitution effects plus 100 largest dominant effects—among the 54,000 estimated effects by Bayes B—was ordered totaling 200 potential QTNs. These effects were reparametrized using the equation in S1 Text. By this equation we weighted the markers effects due to Bayes-B shrinkage specific variance procedure in order to preserve the genotypic values of hybrids in the space of parameter space. It was similar to deregressed breeding value approach.

After to obtain and to reparametrize the 200 genes effects the genomic architecture was constructed based on this QTLs plus null effects distributed over 27,000 markers. Thus, this approach allowed obtaining pleiotropic and linked SNPs along the traits and producing two distinct sources of genetic correlation. The resume of all QTLs are found in the Table 1.

**Table 1:** Counts of coincidence of QTLs identified in the ranking process of the 100 largest additive effects (a) and 100 largest dominance effects (d) obtained in the analysis with the Bayes B model along the five traits.

	EH	EL	ERN	KW
**a**				
**PH**	17	3	1	0
**EH**	-	4	4	2
**EL**	-	-	6	2
**ERN**	-	-	-	1
**d**				
**PH**	20	1	0	2
**EH**	-	1	0	0
**EL**	-	-	0	3
**ERN**	-	-	-	1

Plant height (PH), ear height (EH), ear length (EL), ear row number (ERN) and kernel weight (KW).

In short, each one of five traits presented the following number of QTLs (additive plus dominant effects): 186 (PH), 179 (EH); 200 (EL); 196 (ERN) and 197 KW.

### Building the single-cross hybrids

The artificial crosses were performed using a code developed in the R program in order to obtain 400 single-cross hybrids by using of a 20x20 partial diallel crosses design. The two heterotic groups were defined by graphical analysis based on principal components (Table 2). For this, spectral decomposition was applied on the additive genomic matrix (**A**) related to 4,065 lines and the two PCs were used in the biplot in order to identify the lines more related to B73 and MO17. These two lines present a well-known distinct heterotic pattern.

**Table 2:** Heterotic groups of inbred lines defined by principal component analysis.

Group 1		Group 2	
**B73**	**GEMS-0086**	**Mo17**	**EZ18**
**NC328**	**PI601004**	**Mo44**	**NC44**
**Ames22753**	**PI559382**	**R177**	**Va17**
**PI539927**	**PI539923**	**B97**	**GEMN-0081**
**Ames27151**	**NSL438033**	**GA224**	**IDS91**
**Ames27218**	**GEMS-0223**	**PI601685**	**PI601416**
**PI538009**	**N192**	**A682**	**Ames19008**
**NSL437913**	**PI546485**	**Ames10261**	**Ames19287**
**A679**	**Ames30797**	**Ames27178**	**Ames26764**
**B109**	**PI550473**	**NSL437903**	**PI542778**

The genotypic state for each locus in the hybrid was defined by the expectation of the allelic contribution of each parental line. This expectation was given by:

$$E\left(m_{ij}\right)=\;p_{\lambda j}p_{Ωj}2+p_{\lambda j}\left(1-p_{Ωj}\right)1+p_{Ωj}\left(1-p_{\lambda j}\right)1$$ (2)

where *E*(*m*_*ij*_)is the expectation of the genotypic value at loci j of the hybrid i from the lines λ and *Ω*; *p*_*λj*_ is the favorable allelic frequency in the line λ for the loci j; *p*_*Ωj*_ is the favorable allelic frequency in the line *Ω* for the loci **j**.

For missing markers, intermediated imputed values were observed in line’s SNP matrix, ranging from 0 to 2. Numeric interpolations were applied for missing information through the estimation of allelic frequency for missing markers.

The phenotypic values of hybrids for each of the five traits were built by:

$$y_{SC}=\;\mu +W_{A}a*+W_{D}d*+e$$ (3)

where **y**_**sc**_ is the vector (400×1) of phenotypic value related to single cross hybrids; μ is the general mean related to a specific trait; a* is the vector of allelic substitution effects (27.000×1); d* is the vector of dominance effects (27.000×1); **e** is the residual vector adjusted to different levels of heritability; **W**_**A**_e **W**_**D**_ are the additive and dominant matrices respectively, built from the marker hybrids matrix **M**.

The phenotypic values were adjusted for different levels of heritability (0.3; 0.5 and 0.7). In addition, we used the historic values for each trait. The historical heritability was outlined by [30] and was adapted in the Table 3.

**Table 3:** Mean background heritabilities of five maize traits.

Traits	Heritability (*h*^2^)	Number of studies
**PH**	0.569	45
**EH**	0.662	52
**EL**	0.381	36
**ERN**	0.57	18
**KW**	0.418	11

Plant height (PH); ear height (EH); ear length (EL); ear row number (ERN); kernel weight (KW). Adapted from [8].

The residual vector corrected by a specific heritability was sampled from a normal (0, $\sigma_{e}^{2}$) where $\sigma_{e}^{2}$ is given by:

$$\sigma_{e}^{2}=\frac{\left(1-h^{2}\right)}{h^{2}}\sigma_{g}^{2}$$ (4)

where *h*^2^ is the requested broad-sense heritability and $\sigma_{g}^{2}$ is the genotypic variance (S1 Table).

Thus, the parametric genotypic values are taken as *g* = *μ* +**W**_*A*_a*+**W**_*D*_d*, that this, the model without the simulated error.

### Model Evaluation

Different heritability settings were simulated to test the combined analysis of the five traits with the GBLUP-MV-A and GBLUP-MV-AD models: (i) the five traits in *h*^2^ of 0.3, 0.5, and 0.7; (ii) the traits PH, EH, EL, ERN, KW, in *h*^2^ of 0.3, 0.5, 0.7, 0.7, and 0.3, respectively, randomly sorted by draw; and (iii) the five traits considering the historical *h*^2^ (Table 3). The GBLUP-UV-A and GBLUP-UV-D models were tested with phenotypic data fitted to 0.3, 0.5, and 0.7 heritability, and historical data were tested through individual analysis of the five traits.

The multivariate GBLUP model [17] including the dominance effect can be expressed as:

$$y_{i}=X_{i}\beta_{i}+Z_{i}\alpha_{i}+Z_{i}\delta_{i}+e_{i}$$ (5)

where *y*_*i*_ is the vector of phenotypic observations for trait *i* (*i* = *1*,*2*,*3*,*4*,*5*) of size *n*_*i*_×1, wherein *n*_*i*_ is the total number of observations for trait *i*; *X*_*i*_ is the *n*_*i*_×1 incidence matrix of fixed effects (sample mean) for trait *i*; *Z*_*i*_ is the *n*_*i*_×*n*_*i*_ incidence matrix of random effects (genetic values) for trait *i*, wherein *β*_*i*_ is the mean of the i-^th^ trait, and *α*_*i*_ and *δ*_*i*_ are vectors of allele substitution effects and dominance deviations from *X*_*i*_, *Z*_*i*_, respectively; and *e*_*i*_ are *n*_*i*_×1 residual effects. Because this is an similar to individual animal model, matrix *Z*_*i*_ is an identity matrix and is thus equal for *α*_*i*_ and *δ*_*i*_.

The GBLUP mixed model (5) may be expanded in matrix form as following:

$$\left[\begin{array}{c} y_{1}\\ y_{2}\\ .\\ .\\ .\\ y_{5} \end{array}\right]=\left[\begin{array}{cccccc} X_{1} & O & \ldots & \ldots & \ldots & O\\ O & X_{2} & \ddots & & & \vdots \\ \vdots & \ddots & \ddots & \ddots & & \vdots \\ \vdots & & \ddots & \ddots & \ddots & \vdots \\ \vdots & & & \ddots & \ddots & O\\ O & \ldots & \ldots & \ldots & O & X_{5} \end{array}\right]\left[\begin{array}{c} \beta_{1}\\ \beta_{2}\\ .\\ .\\ .\\ \beta_{5} \end{array}\right]+\left[\begin{array}{cccccc} Z_{1} & O & \ldots & \ldots & \ldots & O\\ O & Z_{2} & \ddots & & & \vdots \\ \vdots & \ddots & \ddots & \ddots & & \vdots \\ \vdots & & \ddots & \ddots & \ddots & \vdots \\ \vdots & & & \ddots & \ddots & O\\ O & \ldots & \ldots & \ldots & O & Z_{5} \end{array}\right]\left[\begin{array}{c} \alpha_{1}\\ \alpha_{2}\\ .\\ .\\ .\\ \alpha_{5} \end{array}\right]+\left[\begin{array}{cccccc} Z_{1} & O & \ldots & \ldots & \ldots & O\\ O & Z_{2} & \ddots & & & \vdots \\ \vdots & \ddots & \ddots & \ddots & & \vdots \\ \vdots & & \ddots & \ddots & \ddots & \vdots \\ \vdots & & & \ddots & \ddots & O\\ O & \ldots & \ldots & \ldots & O & Z_{5} \end{array}\right]\left[\begin{array}{c} \delta_{1}\\ \delta_{2}\\ .\\ .\\ .\\ \delta_{5} \end{array}\right]+\left[\begin{array}{c} e_{1}\\ e_{2}\\ .\\ .\\ .\\ e_{5} \end{array}\right]$$

Thus, it was assumed that **y** ~ *N*(**Xβ**+**Zα**+**Zδ**, **Σ**) where **Σ** = **I** ⊗ **V** and **V** is a 5x5 unstructured matrix and **I** it as *n*×*n* matrix. The prior for random effects **α** and **δ**_*i*_ was assumed as normal distribution given respectively by α~*N*(0,**G**_*α*_) where **G**_*α*_ = **A**⊗**V**_*α*_ and *δ*~*N*(0,**G**_*δ*_) where **G**_*δ*_ = **D**⊗**V**_*δ*_ and where **V**_*α*_ and **V**_*δ*_ are unstructured 5x5matrices.

The additive kinship (**A**) and dominance (**D**) matrices, estimated using molecular markers [31], were obtained using the following equation:

$$A=\frac{W_{A}W_{A}\prime }{2\sum_{k}p_{k}(1-p_{k})}$$ (6)

wherein **p**_**k**_ is the frequency of the favorable allele at locus **k**;

$$D=\frac{W_{D}W_{D}\prime }{4\sum_{k}{\left(p_{k}(1-p_{k})\right)}^{2}}$$ (7)

The matrices obtained by the Kronecker product (⊗) -**R**⊗**I**;**V**_*α*_⊗**A**; ***V***_*δ*_⊗**D**—were entered in Henderson’s mixed model equations (MMEs) [30] according to the specifications of each class of model. The solutions were obtained by direct inversion of the MME after estimating the variance components by maximizing the Residual Maximum Likelihood (REML) function by the Expectation-Maximization (EM) algorithm [32].

Using the EM algorithm described by [32], the REML solution for additive and dominance variance componentes were obtained by:

$${\tilde{\sigma }}_{\alpha_{ij}}=\left[\alpha_{i}^{T}A^{-1}\alpha_{j}+tr\left(AC_{\alpha ij}^{-1}\right)\right]/t$$ (8)

with

$${\tilde{\sigma }}_{\alpha_{ij}}^{}=\{\begin{array}{c} \sigma_{\alpha_{i}}^{2}\;if\;i=j\\ \;\sigma_{\alpha_{ij}}^{}\;otherwise \end{array}$$

and

$${\tilde{\sigma }}_{\delta_{ij}}=\left[\delta_{i}^{T}D^{-1}\delta_{j}+tr\left(DC_{\delta ij}^{-1}\right)\right]/t$$ (9)

$${\tilde{\sigma }}_{\delta_{ij}}^{}=\{\begin{array}{c} \sigma_{\delta_{i}}^{2}\;if\;i=j\\ \;\sigma_{\delta_{ij}}^{}\;otherwise \end{array}$$

where the matrices $C_{\alpha ij}^{-1}$ and $C_{\delta ij}^{-1}$ correspond to submatrices *i j* for additive and dominant effect related to the global mixed models inverse **C**^*-1*^. The residual variance estimator is as follows:

$${\tilde{\sigma }}_{e_{ij}}=\left\{e_{i}^{T}e_{j}+tr\left({\left[WC_{}^{-1}W^{t}\right]}_{ij}\right)\right\}/n$$ (10)

$${\tilde{\sigma }}_{eij}^{}=\{\begin{array}{c} \;\sigma_{e_{k}}^{2}\;if\;i=j\\ \;\sigma_{e_{ij}}^{}\;otherwise \end{array}$$

where **W** is a resultant of columns concatenation related to{***X,Z,Z***}; the trace depends on the submatrices for *i* and *j*; and *n* is the length of the vector {*j*,*i*}.

The component *Z*_*i*_*δ*_*i*_ was discarded when using the additive models for uni and multitrait approaches. In addition, each character *i* was analyzed separately in the case of the univariate model.

### Spectral decomposition

Kinship matrices **A** and **D** can both be non-positive definite; therefore, a spectral decomposition procedure was performed [33,34]. The procedure was basically the decomposition of the kinship matrices into eigenvalue and eigenvector matrices. The negative elements in the diagonal matrix of eigenvalues were replaced by a small positive constant (10^-4^) of decreasing magnitude throughout each diagonal element of the matrix. A positive definite matrix was obtained after the reconstruction, which characterizes the Gaussian process required for the mixed model adopted in this study.

The efficiency of the three models was assessed by three genetic statistics. One was Pearson’s correlation coefficient $r_{g\overset{⌢}{g}}$ between the parametric genetic values *g* and the genetic values estimated $\hat{g}$ by the different models. An additional 10-Fold was used in a cross-validation method to verify the models ability in predicting missing phenotypic data.

The other statistic was the heritability coefficient, estimated by:

$$h_{\hat{g}y_{i}}^{2}=\;\frac{cov\left(\hat{g},y_{i}\right)}{var(y_{i})}$$ (11)

where $\hat{g}$ is only *α*_*i*_ (additive model) or *α*_*i*_+*δ*_*i*_ (additive-dominant model).

The last statistic used was the predicted residual error sum of squares (*PRESS*):

$$PRESS=\;\overset{n_{i}}{\underset{i=1}{\sum }}{\left(\hat{\theta_{i}}-\theta_{i}\right)}^{2}$$ (12)

where $\hat{\theta_{i}}$ is the estimated value of the variables *α*_*i*_ and *δ*_*i*_ (computed separately for each variable) and *θ*_*i*_ the parametric value. The same measure was used for the additive and dominance variance components. All data set and codes used in this work could be accessed in https://github.com/Jhonathan-Pedroso/PlosOne_MVGBLUP_paper

## Results

### Definition of heterotic groups by Principal Component Analysis (PCA)

**Fig 1:**
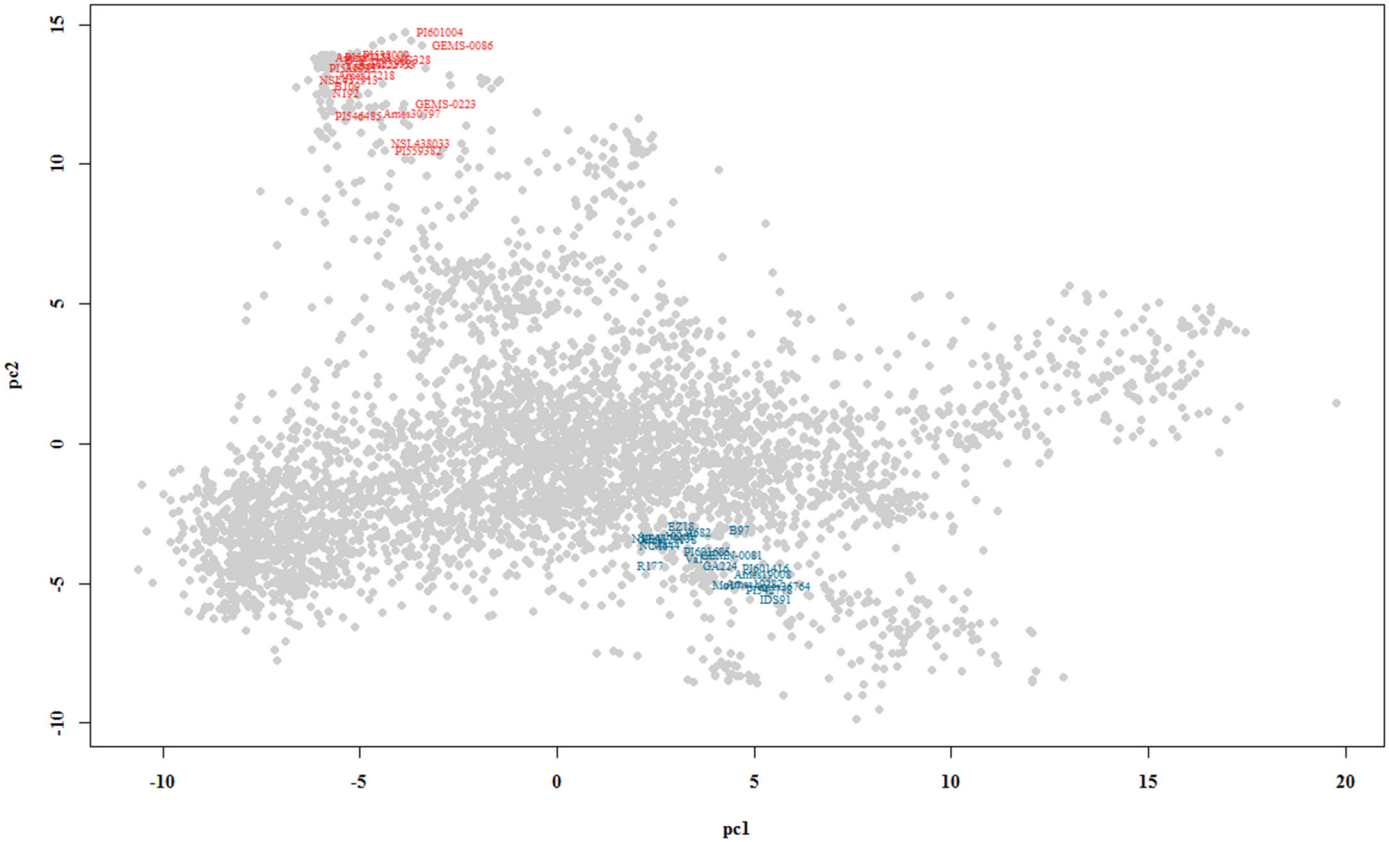
Genetic clustering of 4,091 lines using principal component analysis (PCA). The lines belong to the germplasm bank of the United States Department of Agriculture (USDA). The X-axis represents the first principal component (PC1) and the Y-axis the second principal component (PC2). The colors were assigned according to the genetic group, where blue corresponds to the 19 lines closely related to B73 and red to the 19 lines closely related to Mo17.

A clear distinction between the two genetic groups formed by the lines closely related to B73 and Mo17 can be noted in Fig 1.

The genetic group defined by the lines closely related to B73 was located in the upper left corner of the plot and that closely related to Mo17 was located in the central lower corner. Some lines had replicates in the database, especially the more economically important lines. For example, line B73 had 35 replicates and Mo17 had 7 replicates. As expected, these lines were closely replicated in the plot. The genetic groups were defined in the regions where the highest number of these two reference lines was concentrated.

### Correlations between traits

The additive, dominance and total genetic correlations between traits and parametric values are found in Table 4, S2 and S3 Tables.

**Table 4:** Values of parametric (P) and estimated genetic correlations between five traits using the models GBLUP-UV-A, GBLUP-UV-AD, GBLUP-MV-A and GBLUP-MV-AD in historical heritabilities.

	Model	Effects	EH (0.662)	EL (0.381)	ERN (0.381)	KW (0.418)
**PH**	P	α	0.5501	-0.1310	0.0865	0.2448
		δ	0.5528	0.3302	0.0875	0.3089
		g	0.5477	-0.014	0.0792	0.2665
**EH**		α	-	0.1720	0.5319	0.5583
		δ	-	0.3154	0.0941	0.2364
		g	-	0.2005	0.3400	0.4225
**EL**		α	-	-	0.0550	0.4083
		δ	-	-	0.3040	0.5831
		g	-	-	0.1641	0.4245
**ERN**		α	-	-	-	0.0898
		δ	-	-	-	0.5959
		g	-	-	-	0.2486
**PH**	UV-A	α	0.6266	-0.2848	0.1120	0.2229
**EH**		α	-	-0.0191	0.5607	0.5092
**EL**		α	-	-	0.2064	0.2898
**ERN**		α	-	-	-	0.2898
**PH**	UV-AD	α	0.6787	-0.2502	0.1562	0.2741
		δ	0.4928	0.3554	0.0780	0.3459
		g	0.5731	-0.1066	0.0977	0.2330
**EH**		α	-	-0.0017	0.4497	0.5680
		δ	-	0.2299	0.0736	0.2457
		g	-	0.0439	0.3376	0.4100
**EL**		α	-	-	0.2064	0.2898
		δ	-	-	0.2345	0.2938
		g	-	-	0.2189	0.3372
**ERN**		α	-	-	-	0.2898
		δ	-	-	-	0.1071
		g	-	-	-	0.6239
**PH**	MV-A		0.7602	-0.2442	0.0971	0.4061
**EH**		α	-	-0.0347	0.4422	0.4534
**EL**		α	-	-	0.0418	0.4250
**ERN**		α	-	-	-	-0.0474
**PH**	MV-AD	α	0.7880	-0.2209	0.1902	0.4349
		δ	0.3339	0.3377	-0.0275	0.2769
		g	0.5868	-0.0547	0.1025	0.3692
**EH**		α	-	0.0323	0.3593	0.5447
		δ	-	-0.0991	-0.0217	-0.1155
		g	-	-0.0069	0.2025	0.3545
**EL**		α	-	-	0.0817	0.4432
		δ	-	-	-0.0854	0.8243
		g	-	-	0.0364	0.4954
**ERN**		α	-	-	-	-0.0467
		δ	-	-	-	0.3700
		g	-	-	-	0.0401

Traits: plant height (PH), ear height (EH), ear length (EL), ear row number (ERN), kernel weight (KW). The historical heritability values are in parentheses. The values of genetic correlations between the traits assessed in the process of analysis using the GBLUP-UV model were determined using Pearson’s correlation between the genetic effects estimated in the individual analysis of each trait.

The hybrid construction procedure using the estimates of the additive and dominance genetic effects of the lines of the USDA accessions was effective for the purpose of this study, with correlation values that express what is actually observed. For example, the additive genetic correlation considered parametric (with genetic effects estimated in the lines) between traits PH and EH was 0.5501. This high value is actually expected because tall plants usually have high EH.

There was no clear difference between the models when estimating the correlations and covariance between traits. Despite the small difference between the models, the GBLUP-UV-AD model produced correlation estimates closer to the parametric values considered in the hybrid construction process. For example, the total genetic correlation between traits PH and EH was estimated at 0.5731 using the GBLUP-UV-AD model, while the parametric value was 0.5477.

### Variance components and PRESS

The variance component and PRESS values estimated using the univariate and multivariate models can be found in Table 5, S4 and S5 Tables.

**Table 5:** Estimated and parametric genetic variance components and the sum of squares of the predicted errors of genetic effects estimated using the GBLUP-UV-A, GBLUP-UV-AD, GBLUP-MV-A and GBLUP-MV-AD models in historical heritabilities.

	Model	PH (0.569)	EH (0.662)	EL (0.381)	ERN (0.57)	KW (0.418)
$\sigma_{\alpha }^{2}$	P	23.36	74.65	112.10	0.80	0.35
$\sigma_{\delta }^{2}$		19.07	60.55	33.00	1.00	0.06
$\sigma_{g}^{2}$		42.44	135.20	145.10	1.80	0.41
${\hat{\sigma }}_{\alpha }^{2}$	UV-A	34.86	80.39	159.39	1.23	0.29
*PRESS*_*α*_		986.80	1,476.40	6,544.00	20.00	12.00
*PRESS*_*g*_		7,667.70	24,961.30	18,144.00	388.00	32.00
${\hat{\sigma }}_{\alpha }^{2}$	UV-AD	29.30	86.00	159.22	1.03	0.29
${\hat{\sigma }}_{\delta }^{2}$		23.87	67.99	34.92	0.59	0.05
${\hat{\sigma }}_{g}^{2}$		53.17	154.00	194.14	1.62	0.35
*PRESS*_*α*_		1,117.80	2,008.30	6,802.10	20.70	10.20
*PRESS*_*δ*_		2,461.90	4,251.90	4,473.7	66.70	7.90
*PRESS*_*g*_		2,724.30	5,185.50	9,796.5	70.60	18.8
${\hat{\sigma }}_{\alpha }^{2}$	MV-A	34.47	81.75	154.49	1.20	0.32
*PRESS*_*α*_		1,092.69	1,610.73	6,752.66	23.35	9.89
*PRESS*_*g*_		7,754.87	25,127.55	18,464.66	392.20	33.38
${\hat{\sigma }}_{\alpha }^{2}$	MV-AD	30.11	80.86	153.57	1.02	0.31
${\hat{\sigma }}_{\delta }^{2}$		23.16	66.43	34.54	0.60	0.04
${\hat{\sigma }}_{g}^{2}$		53.27	147.29	188.11	1.61	0.35
*PRESS*_*α*_		1,322.95	2,406.62	7,158.72	22.62	9.76
*PRESS*_*δ*_		2,585.86	4,130.27	4,783.29	69.59	10.17
*PRESS*_*g*_		2,810.18	4,937.93	10,354.14	71.52	20.98

Estimated additive (${\hat{\sigma }}_{\alpha }^{2}$), dominance (${\hat{\sigma }}_{\delta }^{2}$) and total (${\hat{\sigma }}_{g}^{2}$) genetic variance and sum of squares of additive (*PRESS*_*α*_), dominance (*PRESS*_*δ*_) and total genetic (*PRESS*_*g*_) predicted residual errors for the traits plant height (PH), ear height (EH), ear length (EL), ear row number (ERN), and kernel weight (KW). The background heritability values are in parentheses.

Marginal differences in the efficiency of the GBLUP-UV-A and GBLUP-MV-A models were detected in any heritability settings when estimating the additive genetic variance or between the GBLUP-UV-AD and GBLUP-MV-AD models when estimating the additive (${\hat{\sigma }}_{\alpha }^{2}$), dominance (${\hat{\sigma }}_{\delta }^{2}$) and total (${\hat{\sigma }}_{g}^{2}$) genetic variance. Small superiority fluctuations were observed between the models depending on the trait. However, because of their small magnitude, these variations were not sufficiently informative to suggest superiority between models. The similar values of additive genetic variance components obtained using the additive and additive-dominant models suggest some orthogonality in the decomposition of the variance components in most of the genetic architectures studied. However, confounding between the additive genetic variance and the dominance genetic variance was observed in the additive models in high-heritability scenarios. For example, the ${\hat{\sigma }}_{\alpha }^{2}$ variance values estimated using the GBLUP-UV-A model for trait PH fitted to 0.3, 0.5 and 0.7 heritability were 30.99, 43.13 and 51.45, respectively, while the parametric value was 23.36. This result evidences the greater absorption of dominance genetic variance into the additive genetic variance component in superior heritability scenarios when using models exclusively considering additive effects.

Although the latter result is apparently paradoxical because increased efficiency in the analysis of traits is usually expected under conditions of high heritability, we know that according to quantitative genetics theory, both the additive and dominance genetic variances are functions of the dominance effect of markers:

$$\sigma_{\alpha }^{2}=\sum_{\text{k}}2p_{k}(1-p_{k}){\left[a_{k}+d_{k}(q_{k}-p_{k})\right]}^{2}\;;\;\sigma_{\delta }^{2}={\sum_{\text{k}}\left(2p_{k}(1-p_{k})d_{k}\right)}^{2}$$

Accordingly, both the additive and dominance genetic variance components are theoretically expected to exhibit strong confounding when either one or the other is not fitted to the model.

The PRESS values of the total genetic effects obtained using the GBLUP-MV-A model were higher than the values resulting from the GBLUP-UV-A model, and marginal differences were found between the GBLUP-MV-AD and GBLUP-UV-AD models. Considerable reductions in PRESS were observed only when including the dominance effects in the model. The most relevant reduction was observed in the analysis of trait EH in the historical heritability scenario using the GBLUP-MV-AD model, wherein there was a reduction in the PRESS of the total genetic effects from 25,127.55 to 4,937.93 when including the dominance effect in the model (Table 4). Reductions in the PRESS of total genetic effects and small variations without the pattern of the PRESS of additive genetic effects were observed in all analysis scenarios when including the dominance effect. These results evidence the importance of including the dominance effects in models during the analysis of traits of maize hybrids.

### Predictive ability

**Fig 2:**
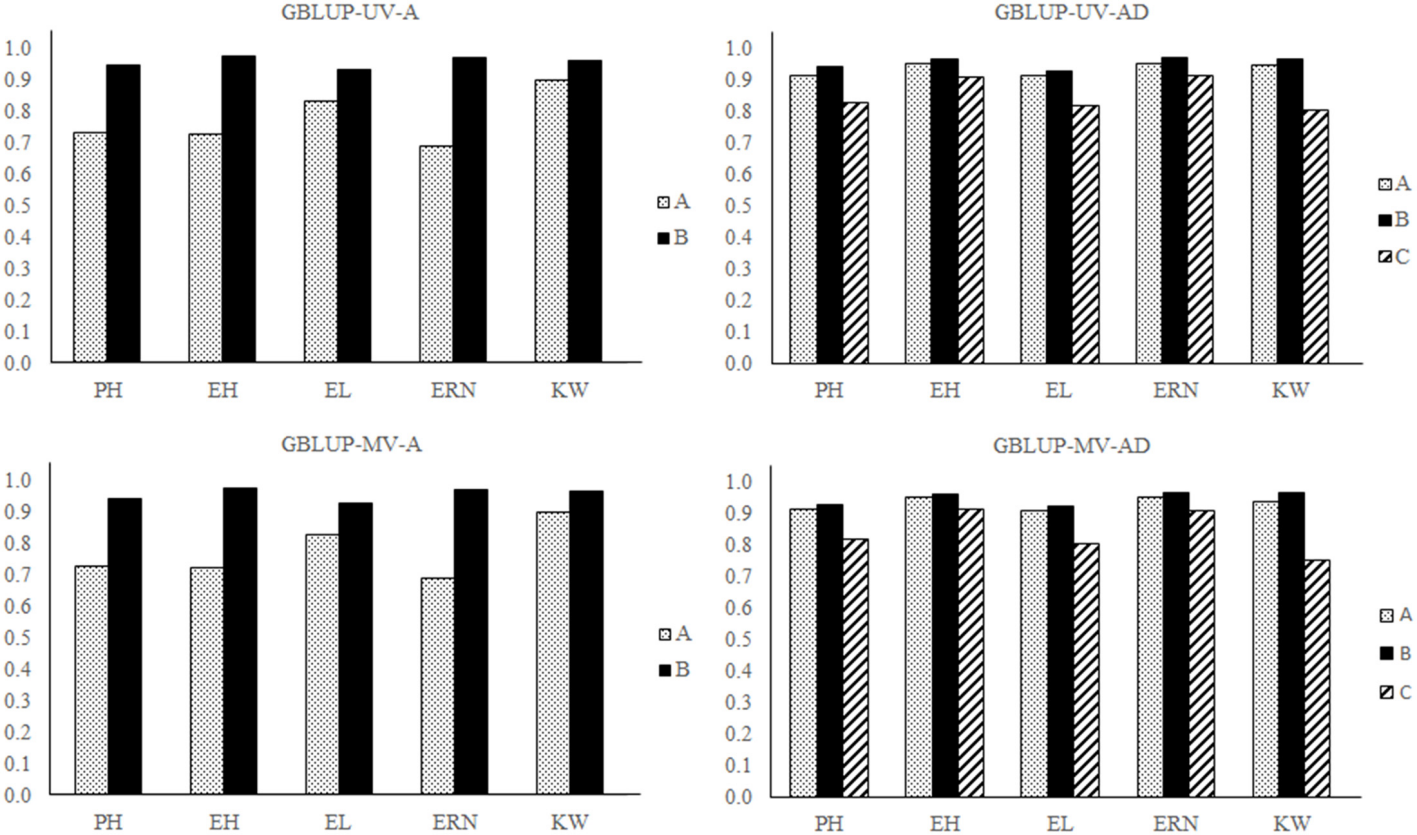
Correlations between the parametric and estimated genetic effects of the traits fitted to background heritabilities. Models used: GBLUP-UV-A, GBLUP-UV-AD, GBLUP-MV-A and GBLUP-MV-AD. Traits analyzed: Plant height (PH), ear height (EH), ear length (EL), ear row number (ERN) and kernel weight (KW). (A) correlations between the parametric and estimated total genetic effects; (B) correlations between the parametric and estimated additive effects; (C) correlations between the parametric and estimated dominance effects.

The correlations between the parametric and estimated genetic values in the analysis of data fitted to background heritability scenarios are shown in Fig 2.

No clear difference in predictive ability was also observed between the univariate and multivariate models in any scenario tested (Fig 2, S1–S4 Figs). However, considerable differences were observed between the additive and additive-dominant models. For example, the total genetic correlation in the analysis of trait EH fitted to background heritability using the GBLUP-UV-AD model was 38.91% higher than that obtained using the GBLUP-UV-A model; in the GBLUP-MV-AD model, it was 37.94% higher than the GBLUP-MV-A model. The correlations were virtually identical between parametric and estimated additive genetic effects in all models tested. This same pattern of correlations was observed in all dominance genetic effects.

**Fig 3:**
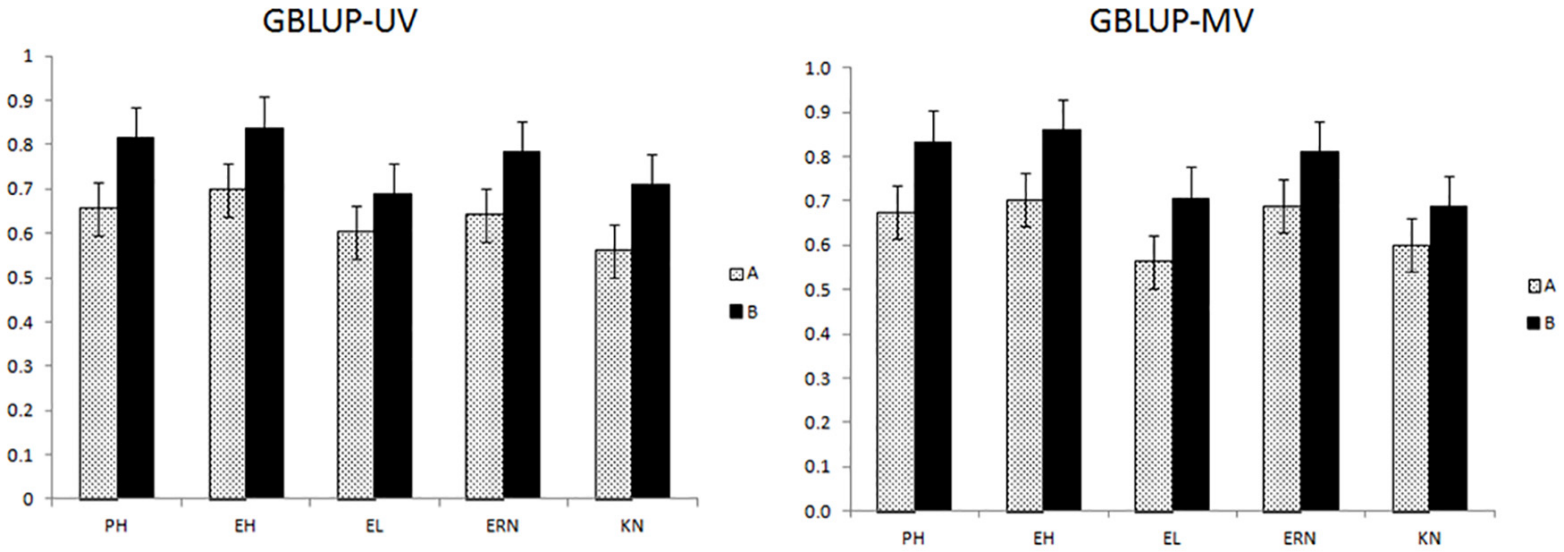
Correlations between the predicted and observed values based in a 10-fold cross-validation traits fitted to background heritabilities. Models used: GBLUP-UV-A, GBLUP-UV-AD, GBLUP-MV-A, and GBLUP-MV-AD. Traits analyzed: Plant height (PH), ear height (EH), ear length (EL), ear row number (ERN) and kernel weight (KW).

In relation to k-fold results, there was a marginal difference between GBLUP-UV and GBLUP-MV (Fig 3). Once again, the inclusion of dominance effect makes the model perform better than the additive one. The average correlation for GBLUP-A was 0.632 and 0.647 for uni (UV) and multivariate (MV) scenarios. For GBLUP AD, the correlations were of 0.766 and 0.779 for UV and MV, respectively. The standard deviations related to k-fold using the GBLUP-UV-A and GBLUP-MV-A were very similar (0.06). For the dominance model, the k-fold standard deviations were about 0.07 and 0.075 for GBLUP-UV-AD and GBLUP-MV-AD respectively.

### Heritability coefficients

**Fig 4:**
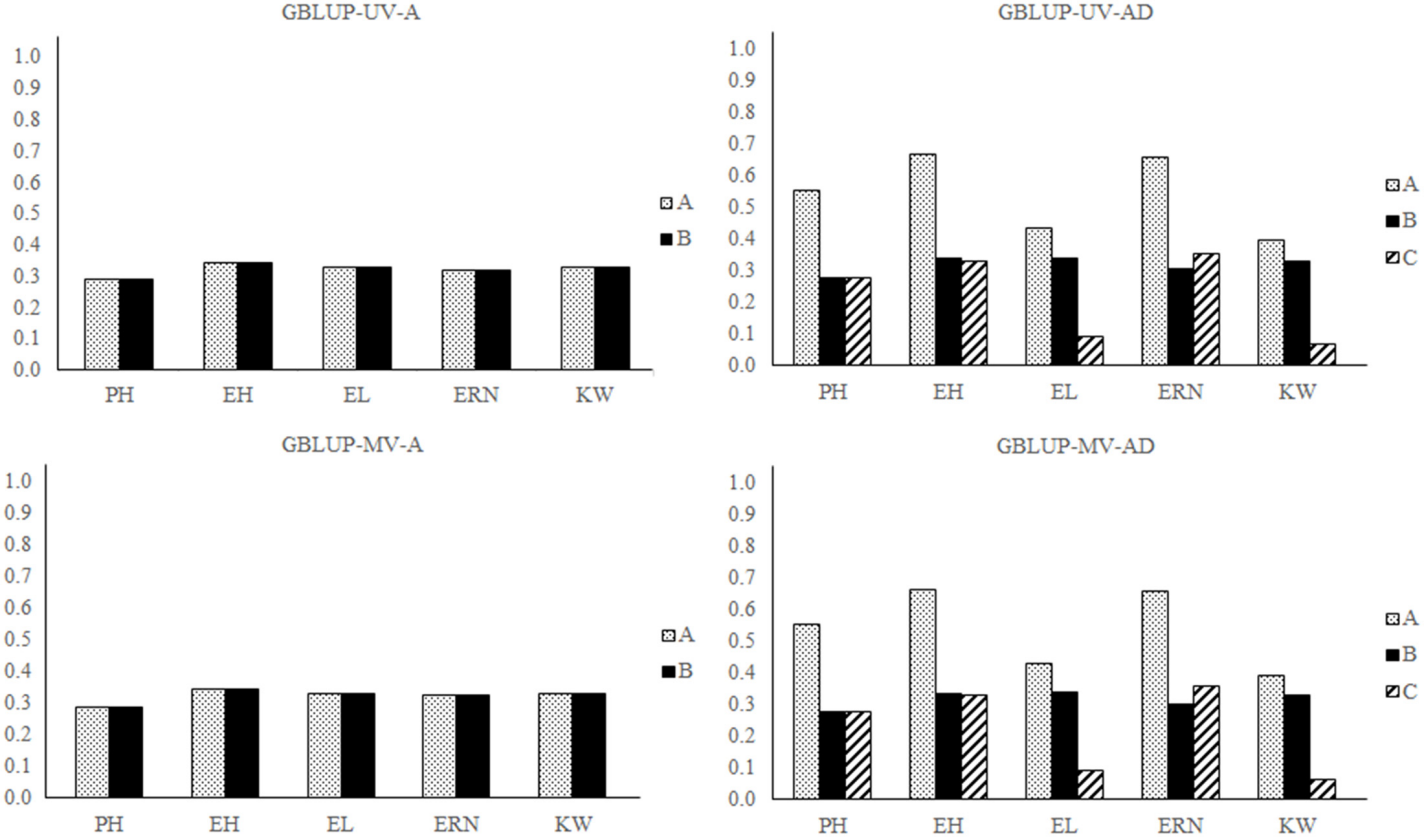
Heritability coefficients of traits fitted to background heritability. Models used: GBLUP-UV-A, GBLUP-UV-AD, GBLUP-MV-A and GBLUP-MV-AD. Traits analyzed: Plant height (PH), ear height (EH), ear length (EL), ear row number (ERN) and kernel weight (KW). (A) Broad-sense heritability coefficients; (B) Additive heritability coefficients; (C) Dominance heritability coefficients.

The heritability coefficient estimates obtained in all models, in all scenarios of analysis, are shown in S5–S8 Figs and Fig 4.

The same pattern of lack of difference in performance between the multivariate and univariate models was observed for the heritability estimates. The superiority of the models including the dominance effect is also clearly shown by the amount of total genetic data recovered. The smallest impact of including the dominance effect in background heritability scenarios was observed when using the GBLUP-MV-AD model in the analysis of trait KW, in which there was a 17.85% increase in broad-sense heritability compared to the GBLUP-MV-A model. However, the GBLUP-MV-AD model exhibited the highest ability to recover genetic data when including the dominance effect. There was a broad-sense heritability increase from 0.3228 to 0.6584 in the analysis of trait ERN using this model (Fig 4). These results confirm the superiority of models that include dominance genetic effects in recovering genetic data but showed a small difference between univariate and multivariate models in the genetic architecture scenarios addressed.

## Discussion

In this study, several genetic architecture scenarios with variations in heritability conditions were created to evaluate the usefulness of multivariate models in maize genetic breeding programs, primarily seeking to analyze background genetic architecture conditions previously reported in other scientific publications. This is the first study to evaluate the usefulness of the multivariate GBLUP model in analyses of production components, particularly in maize. Studies evaluating the performance of multivariate models with data on molecular markers have already been reported. However, the impact of including the dominance effect in prediction processes is reported herein.

The use of genetic data on the NAM population (4,091 inbred lines) enabled the exploration of a gene pool rarely found in a genetic breeding program restricted to one company or university. The high genetic variability of that population helped to estimate the genetic parameters representative of several germplasm sources. Genetic parameter scenarios based on background results were also adopted by [18], who analyzed actual and simulated data on common bean traits using multivariate models. However, that study did not evaluate the impact of including kinship data estimated by molecular markers and including dominance effects. Most studies with simulations take on extreme scenarios wherein the traits of interest may not be found. For example, a previous study [22] adopted 0.1, 0.5 and 0.8 heritability conditions and evaluated only two traits together. Therefore, we sought to evaluate heritability conditions commonly observed during the genetic breeding of maize according to background values [35]. Regarding the correlations generated by our process of artificial crosses, the high value of total genetic correlation between PH and EH (0.5477) is clear evidence of the successful construction of the genome of the hybrids without requiring an unrealistic simulation process with genetic correlations between the traits generated from genetic effects obtained by sampling processes. In the genomic analysis of the lines, we identified 30 QTLs with pleiotropic effects—10 QTLs with additive effects, 13 QTLs with dominance effects and 7 QTLs with additive and dominance effects—that act on the genetic control of traits PH and EH. A median genetic correlation of 0.4225 occurred between traits EH and KW, which had two QTLs with pleiotropic additive effects. The striking impact of QTLs with pleiotropic effects on the origin of the correlations between the traits is also evidenced by the small genetic correlation value of 0.0762 between traits PH and ERN, which only had one QTL with additive pleiotropic effect. This pattern of genetic correlations generated by pleiotropic QTLs was also observed between the other phenotypic traits of simple hybrids constructed by our process of artificial crosses. Thus, based on the results of the strategy adopted, we suggest that phenotypic observations designed based on genetic data from open-access databases are alternatives that enable the gathering of data from artificial crosses maintaining the genetic architecture of quantitative traits. These data can be reused for testing and developing comparative studies and proposals of statistical-genetic models.

### Univariate GBLUP versus multivariate GBLUP

The main factors already reported that may affect the predictive performance during the use of multivariate models with kinship data are (i) the availability of phenotypic traits well correlated with the target trait; (ii) good genome coverage using molecular markers; (iii) low/moderate heritability of the target trait; (iv) high heritability of the traits correlated with the target; and (v) moderate/high correlation between traits [18,21].

Our results diverged partly from those reports. There were no improvements in predictive ability between PH and KW in the analyses using multivariate models for traits PH, EH, EL, ERN and KW fitted to 0.3, 0.5, 0.7, 0.7 and 0.3 heritability, respectively. This result was not expected because both traits have low heritability and are moderately correlated with the others.

The results of the analysis of data generated in historical heritability scenarios also show no clear differences between the multivariate and univariate GBLUP models. This fact corroborates the findings reported in other studies, wherein combined analyses of traits using multivariate models tend to have a predictive performance similar to univariate models under intermediate conditions of heritability and correlation [18,21,22].

No advantage resulted from adopting the multivariate model under the historical scenario given the greater computational demand required by the multivariate method and the marginal difference with the univariate methods. The same result was observed by [36] in GS. However, those authors observed that differences between univariate and multivariate genomic models only occur in unbalanced data scenarios, and the multivariate model was recommended in this only specific case. In our work we do not considered unbalanced data, but this could be an issue for further studies.

Situations in which all or most traits have low heritability [18] are among the conditions that may have a detrimental impact on the performance of multivariate analysis. S1 Fig shows that the analysis of the five traits fitted to 0.3 heritability caused no differences in predictive ability between the univariate and multivariate models. Our results suggest that the multivariate analysis of traits under conditions of low/moderate correlation with heritability of up to 0.3 are not detrimental compared to the univariate analyses. Another important observation is the lack of benefit from the multivariate analysis using all five traits fitted to a fixed heritability of 0.7 (S3 Fig). This result suggests that the combined analysis of all traits at high heritability also offers no benefit over the individual analysis under conditions of low/moderate correlation between traits.

The correlations of total genetic effects between traits ranged from -0.014 between traits PH and EL to 0.5477 between traits PH and EH in the analysis of data from the 4,091 inbred lines from the Panzea database. A simulation study [21] showed that the multivariate analysis is only advantageous in scenarios of correlation between traits higher than 0.5 in some specific genetic architecture constructions. This finding may be a plausible explanation for the lack of advantage from using the multivariate GBLUP model examined in our study because low/moderate correlations were observed between the genetic values of the traits of the lines analyzed.

The adoption of indirect selection in maize could be not always advantageous compared to direct selection for grain yield. Previously published studies have shown that despite the existence of traits well correlated with grain yield, these secondary traits usually do not exhibit high heritability encouraging the adoption of indirect selection to increase the selection gains, and when there is high heritability, high correlation may not occur [35,37]. We believe this may be the same explanation for the small discrepancy between the results obtained using the univariate and multivariate models in the analysis of the five maize traits studied, as we only observed low/moderate correlation and moderate heritability between the genetic effects of traits.

### Inclusion of dominance effects

The use of multivariate models adopting mixed models was first proposed by [17]. The proposal of including non-additive effects in univariate models with kinship data estimated by pedigrees was proposed by [38]. In that study, C. R. Henderson had already suggested the possibility of including non-additive effects in multivariate models. Studies using those models were not immediately conducted given the computational constraints existing at the time.

Several applied prediction studies including non-additive effects have been conducted. Among these studies, [39] examined the ability of the models to break down the genetic variance components considering only data on molecular markers and using data on *Pinus taeda* pedigrees alone with univariate models. The main conclusion was that molecular markers are more informative for estimating the kinship matrices of additive and non-additive effects and that they enable the orthogonal decomposition of the variance components. Those authors also argue that kinship matrices of non-additive effects designed by pedigree have serious restrictions that may compromise inference processes. The explanation is that all elements of these matrices are functions of the elements of the additive kinship matrix and, therefore, are highly correlated with the elements of other kinship matrices, thereby precluding the effective decomposition of variance components. Because of this assertion, we did not compare data on pedigrees versus molecular markers. Using only molecular markers, we concluded that both the GBLUP-UV-AD and GBLUP-MV-AD models effectively decompose the total genetic variance components into additive and dominance components, especially in low-to-moderate heritability scenarios. A previous study [40] reported that the difference in predictive ability between the additive and additive-dominant models is significant under low-heritability conditions and disappears under high-heritability conditions. Although we did not observe the same trend in predictive ability, greater absorption of the dominant variance component into the additive variance in models whose dominance deviation is ignored tends to occur under high-heritability conditions. The confounding between variance components may encourage incorrect conclusions, which may lead to detrimental decisions in breeding programs, especially in populations with some level of selection (*p* ≠ 0.05).

Among the available kinship matrices, we chose to use the one proposed by [31]. This matrix allows orthogonality in the decomposition of genetic variance components [28,41,42]. The inclusion of epistatic effects was not examined in this study. However, new studies seeking alternative procedures to estimate epistatic effects and to evaluate the impact of including these co-variables in multivariate models are necessary.

A considerable increase in the values of correlations between parametric and simulated genetic effects was observed when including the dominance effects in the GBLUP-UV and GBLUP-MV models. This impact has already been reported in other studies using the GBLUP-UV model [30,39,40,43]. In addition to improving the predictive ability of these models, the inclusion of dominance effects improved the overall quality of the estimation of additive genetic variance components, given the confounding already reported between these components in purely additive models in a study of genomic selection models [43].

Also, in cross-validation results, the inclusion of the dominance improved the correlation between missing and predicted values about 20% indicating its importance to predict missing values. On the other hand, there was no advantage of the multivariate over the univariate model for cross-validation data. It is worth to highlight that in cross-validation results, the accuracy threshold can be given by square root of heritability. In this sense, the accuracies from the cross-validation process were slightly lower than those obtained by true vs. predicted values. It can be questioned whether cross-validation is necessary given we know the true values. In a real situation, we do not know the true values and cross-validation might be a useful tool to evaluate the predictive model. Therefore, using the dominance effect, it was observed that the cross-validation predictions reached the heritability limits (approximately 0.75) exploring all genetic variation. Because of the greater computational demand required by the multivariate method and the marginal difference with the univariate methods, there was no advantage in adopting the multivariate model under a simulated historical scenario and using balanced data. The same result was observed by [36] in GS when these authors compared the GBLUP-UV-A and GBLUP-MV-A models. However, those authors only observed differences in unbalanced data scenarios, and the multivariate model was better in this specific case.

## Conclusions

Our strategy of constructing single-cross hybrids using real data on maize lines was effective at preserving the genetic architecture of the traits tested. There were no benefits related to predictive ability or to the quality of the decomposition of variance components in the analysis of the traits plant height, ear height, ear length, ear row number and kernel weight of maize when adopting the multivariate GBLUP compared to the univariate GBLUP. There were considerable gains in predictive ability and quality of the decomposition of variance components when dominance effects were included in both the univariate and multivariate analyses using the GBLUP model for the traits studied.

## Supporting Information

S1 Fig Correlations between the parametric and estimated genetic effects of the traits fitted to the fixed heritability of 0.3. Models used: GBLUP-UV-A, GBLUP-UV-AD, GBLUP-MV-A and GBLUP-MV-AD. Traits analyzed: Plant height (PH), ear height (EH), ear length (EL), ear row number (ERN) and kernel weight (KW). (A) correlations between the parametric and estimated total genetic effects; (B) correlations between the parametric and estimated additive effects; (C) correlations between the parametric and estimated dominance effects.
(TIF)

S2 Fig Correlations between the parametric and estimated genetic effects of the traits fitted to the fixed heritability of 0.5. Models used: GBLUP-UV-A, GBLUP-UV-AD, GBLUP-MV-A and GBLUP-MV-AD. Traits analyzed: Plant height (PH), ear height (EH), ear length (EL), ear row number (ERN) and kernel weight (KW). (A) correlations between the parametric and estimated total genetic effects; (B) correlations between the parametric and estimated additive effects; (C) correlations between the parametric and estimated dominance effects.
(TIF)

S3 Fig Correlations between the parametric and estimated genetic effects of the traits fitted to the fixed heritability of 0.7. Models used: GBLUP-UV-A, GBLUP-UV-AD, GBLUP-MV-A and GBLUP-MV-AD. Traits analyzed: Plant height (PH), ear height (EH), ear length (EL), ear row number (ERN) and kernel weight (KW). (A) correlations between the parametric and estimated total genetic effects; (B) correlations between the parametric and estimated additive effects; (C) correlations between the parametric and estimated dominance effects.
(TIF)

S4 Fig Correlations between the parametric and estimated genetic effects of the traits fitted at random to the heritability of 0.3 (PH and KW); 0.5 (EH) and 0.7 (EL and ERN). Models used: GBLUP-MV-A and GBLUP-MV-AD. Traits analyzed: Plant height (PH), ear height (EH), ear length (EL), ear row number (ERN) and kernel weight (KW). (A) correlations between the parametric and estimated total genetic effects; (B) correlations between the parametric and estimated additive effects; (C) correlations between the parametric and estimated dominance effects.
(TIF)

S5 Fig Estimated heritability coefficients of the traits fitted to the fixed heritability of 0.3. Models used: GBLUP-MV-A and GBLUP-MV-AD. Traits analyzed: Plant height (PH), ear height (EH), ear length (EL), ear row number (ERN) and kernel weight (KW). (A) Broad-sense heritability coefficients; (B) Additive heritability coefficients; (C) Dominance heritability coefficients.
(TIF)

S6 Fig Estimated heritability coefficients of the traits fitted to the fixed heritability of 0.5. Models used: GBLUP-UV-A, GBLUP-UV-AD, GBLUP-MV-A and GBLUP-MV-AD. Traits analyzed: Plant height (PH), ear height (EH), ear length (EL), ear row number (ERN) and kernel weight (KW). (A) Broad-sense heritability coefficients; (B) Additive heritability coefficients; (C) Dominance heritability coefficients.
(TIF)

S7 Fig Estimated heritability coefficients of the traits fitted to the fixed heritability of 0.7. Models used: GBLUP-UV-A, GBLUP-UV-AD, GBLUP-MV-A and GBLUP-MV-AD. Traits analyzed: Plant height (PH), ear height (EH), ear length (EL), ear row number (ERN) and kernel weight (KW). (A) Broad-sense heritability coefficients; (B) Additive heritability coefficients; (C) Dominance heritability coefficients.
(TIF)

S8 Fig Estimated heritability coefficients of the traits fitted at random to the heritability of 0.3 (PH and KW); 0.5 (EH) and 0.7 (EL and ERN). Models used: GBLUP-MV-A e GBLUP-MV-AD. Traits analyzed: Plant height (PH), ear height (EH), ear length (EL), ear row number (ERN) and kernel weight (KW). (A) Broad-sense heritability coefficients; (B) Additive heritability coefficients; (C) Dominance heritability coefficients.
(TIF)

S1 Table Additive, dominant and total genetic variance components obtained by the analysis of the inbred lines. These parameters were considered as true during the building of the single-cross hybrids. Traits: plant height (PH), ear height (EH), ear length (EL), ear row number (ERN), kernel weight (KW).
(DOCX)

S2 Table Additive correlations estimated between the five traits using GBLUP-MV-A. Traits: plant height (PH), ear height (EH), ear length (EL), ear row number (ERN), kernel weight (KW). The adjusted heritability values used during the data construction are in parentheses.
(DOCX)

S3 Table Additive, dominant and total genetic correlations estimated between the five traits using GBLUP-MV-AD. Traits: plant height (PH), ear height (EH), ear length (EL), ear row number (ERN), kernel weight (KW). The adjusted heritability values used during the data construction are in parentheses.
(DOCX)

S4 TableEstimated and parametric genetic variance components and the sum of squares of the predicted errors of genetic effects estimated using the GBLUP-UV-A and GBLUP-UV-AD.Estimated additive ($\sigma_{\alpha }^{2}$), dominance ($\sigma_{\delta }^{2}$) and total ($\sigma_{g}^{2}$) genetic variance and sum of squares of additive (*PRESS*_α_), dominance (*PRESS*_δ_) and total genetic (*PRESS_g_*) predicted residual errors for the traits plant height (PH), ear height (EH), ear length (EL), ear row number (ERN), and kernel weight (KW). The adjusted heritability values used during the data construction are in parentheses.(DOCX)

S5 TableEstimated and parametric genetic variance components and the sum of squares of the predicted errors of genetic effects estimated using the GBLUP-MV-A and GBLUP-MV-AD.Estimated additive ($\sigma_{\alpha }^{2}$), dominance ($\sigma_{\delta }^{2}$) and total ($\sigma_{g}^{2}$) genetic variance and sum of squares of additive (*PRESS*_α_), dominance (*PRESS*_δ_) and total genetic (*PRESS_g_*) predicted residual errors for the traits plant height (PH), ear height (EH), ear length (EL), ear row number (ERN), and kernel weight (KW). The adjusted heritability values used during the data construction are in parentheses.(DOCX)

S1 Text Weighting additive and dominant values for the markers. (DOCX)
