# Supplementary figures and images for: Inclusion of Dominance Effects in the Multivariate GBLUP Model
Source: PLoS One. 2016 Apr 13;11(4):e0152045. doi: 10.1371/journal.pone.0152045 (PMC4830534; doi:10.1371/journal.pone.0152045)

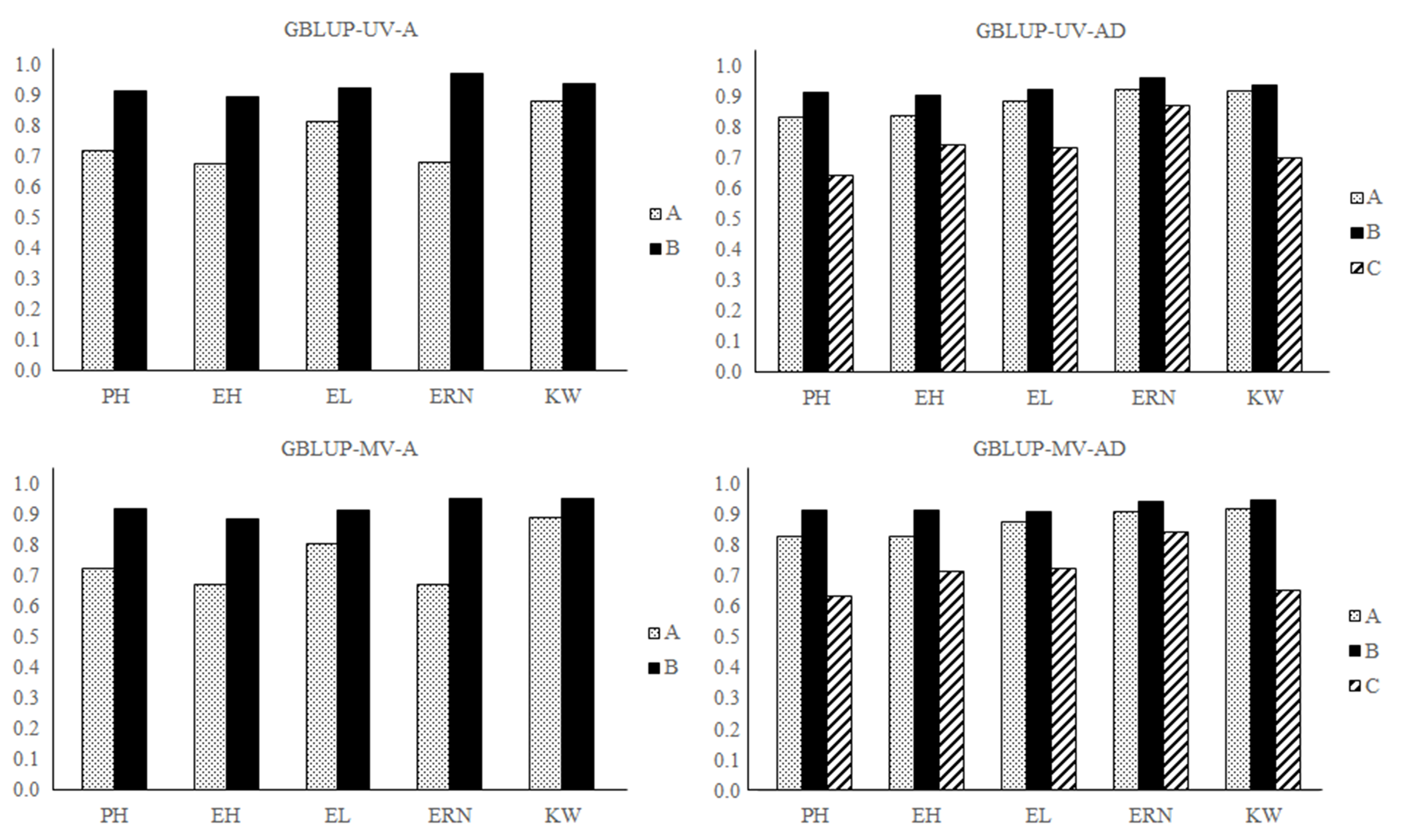

Supplement: S1 Fig — Models used: GBLUP-UV-A, GBLUP-UV-AD, GBLUP-MV-A and GBLUP-MV-AD. Traits analyzed: Plant height (PH), ear height (EH), ear length (EL), ear row number (ERN) and kernel weight (KW). (A) correlations between the parametric and estimated total genetic effects; (B) correlations between the parametric and estimated additive effects; (C) correlations between the parametric and estimated dominance effects. (TIF) [file pone.0152045.s001.tif]

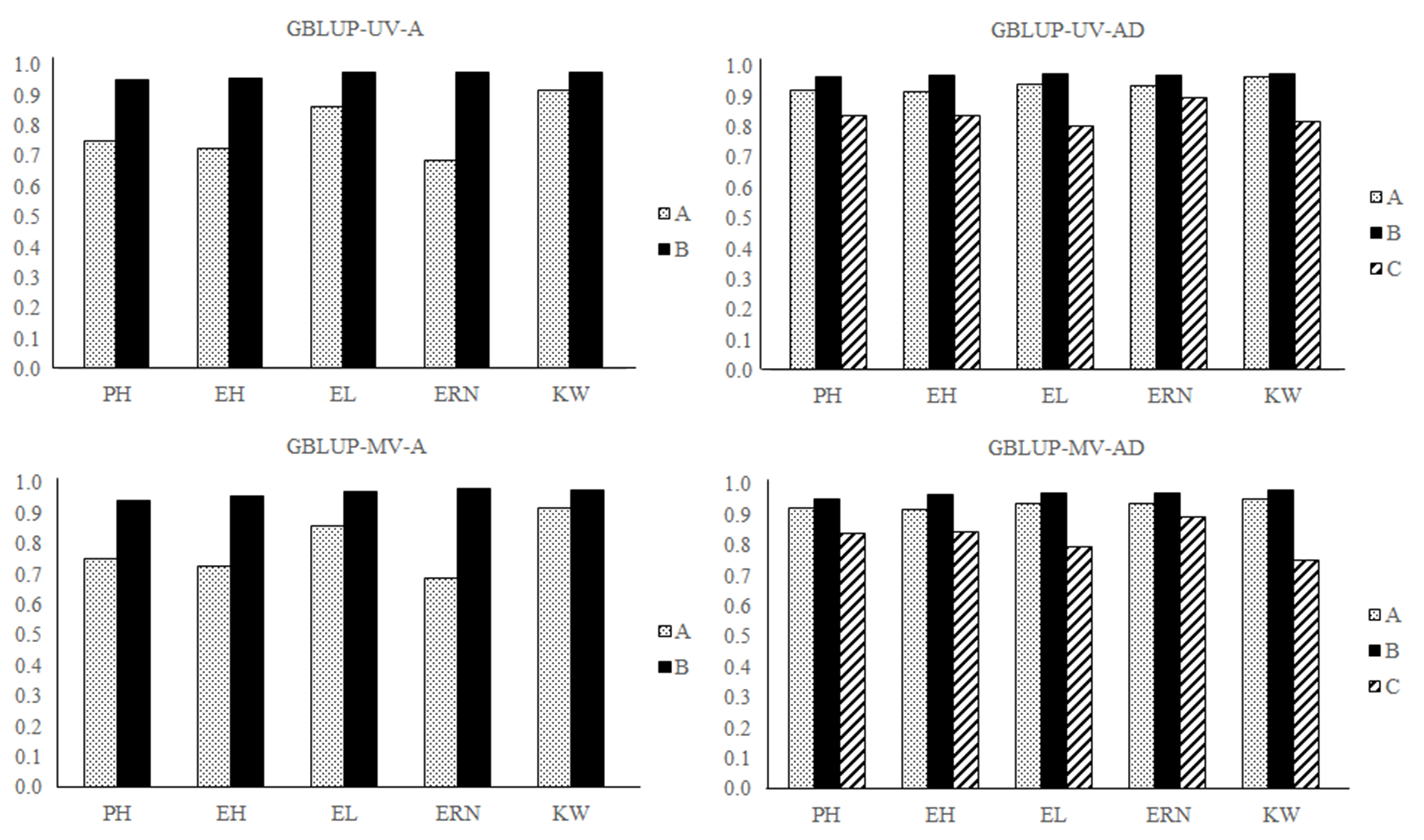

Supplement: S2 Fig — Models used: GBLUP-UV-A, GBLUP-UV-AD, GBLUP-MV-A and GBLUP-MV-AD. Traits analyzed: Plant height (PH), ear height (EH), ear length (EL), ear row number (ERN) and kernel weight (KW). (A) correlations between the parametric and estimated total genetic effects; (B) correlations between the parametric and estimated additive effects; (C) correlations between the parametric and estimated dominance effects. (TIF) [file pone.0152045.s002.tif]

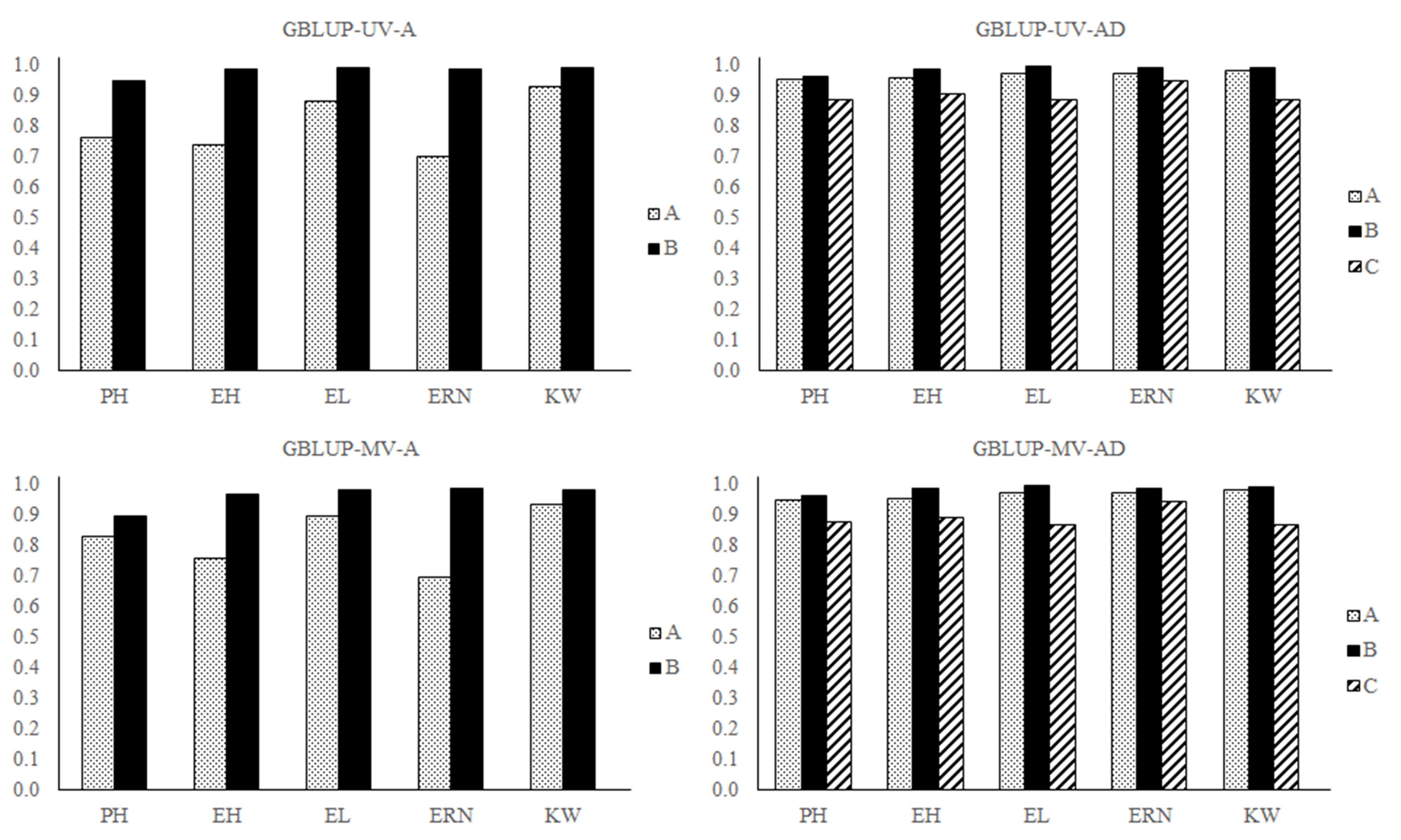

Supplement: S3 Fig — Models used: GBLUP-UV-A, GBLUP-UV-AD, GBLUP-MV-A and GBLUP-MV-AD. Traits analyzed: Plant height (PH), ear height (EH), ear length (EL), ear row number (ERN) and kernel weight (KW). (A) correlations between the parametric and estimated total genetic effects; (B) correlations between the parametric and estimated additive effects; (C) correlations between the parametric and estimated dominance effects. (TIF) [file pone.0152045.s003.tif]

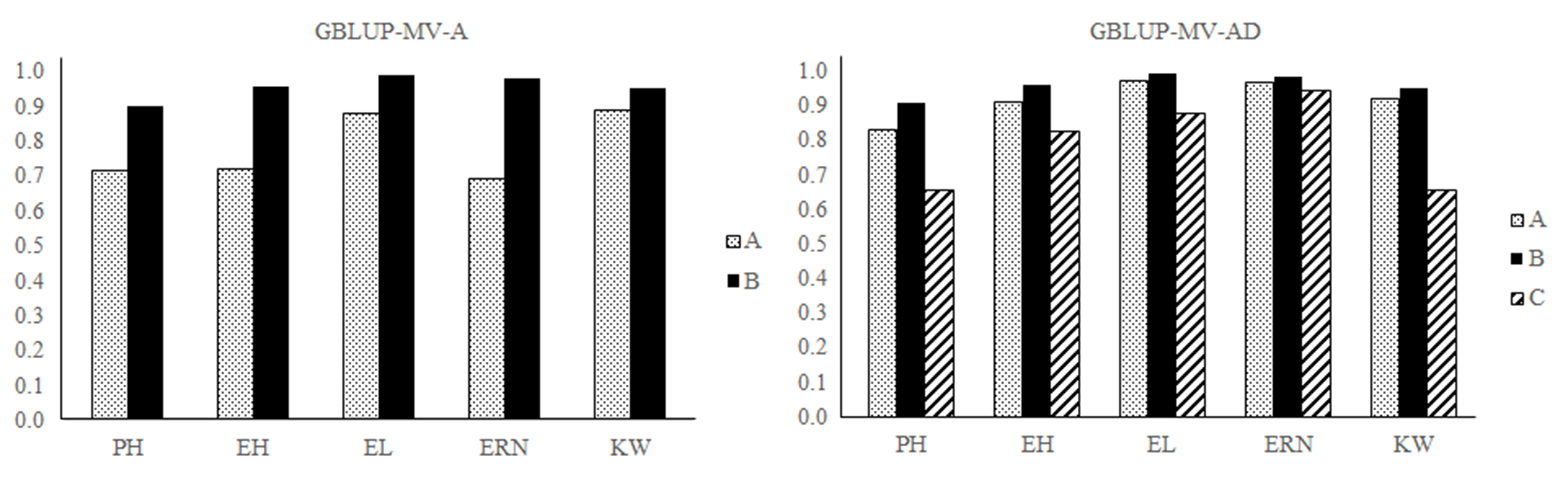

Supplement: S4 Fig — Models used: GBLUP-MV-A and GBLUP-MV-AD. Traits analyzed: Plant height (PH), ear height (EH), ear length (EL), ear row number (ERN) and kernel weight (KW). (A) correlations between the parametric and estimated total genetic effects; (B) correlations between the parametric and estimated additive effects; (C) correlations between the parametric and estimated dominance effects. (TIF) [file pone.0152045.s004.tif]

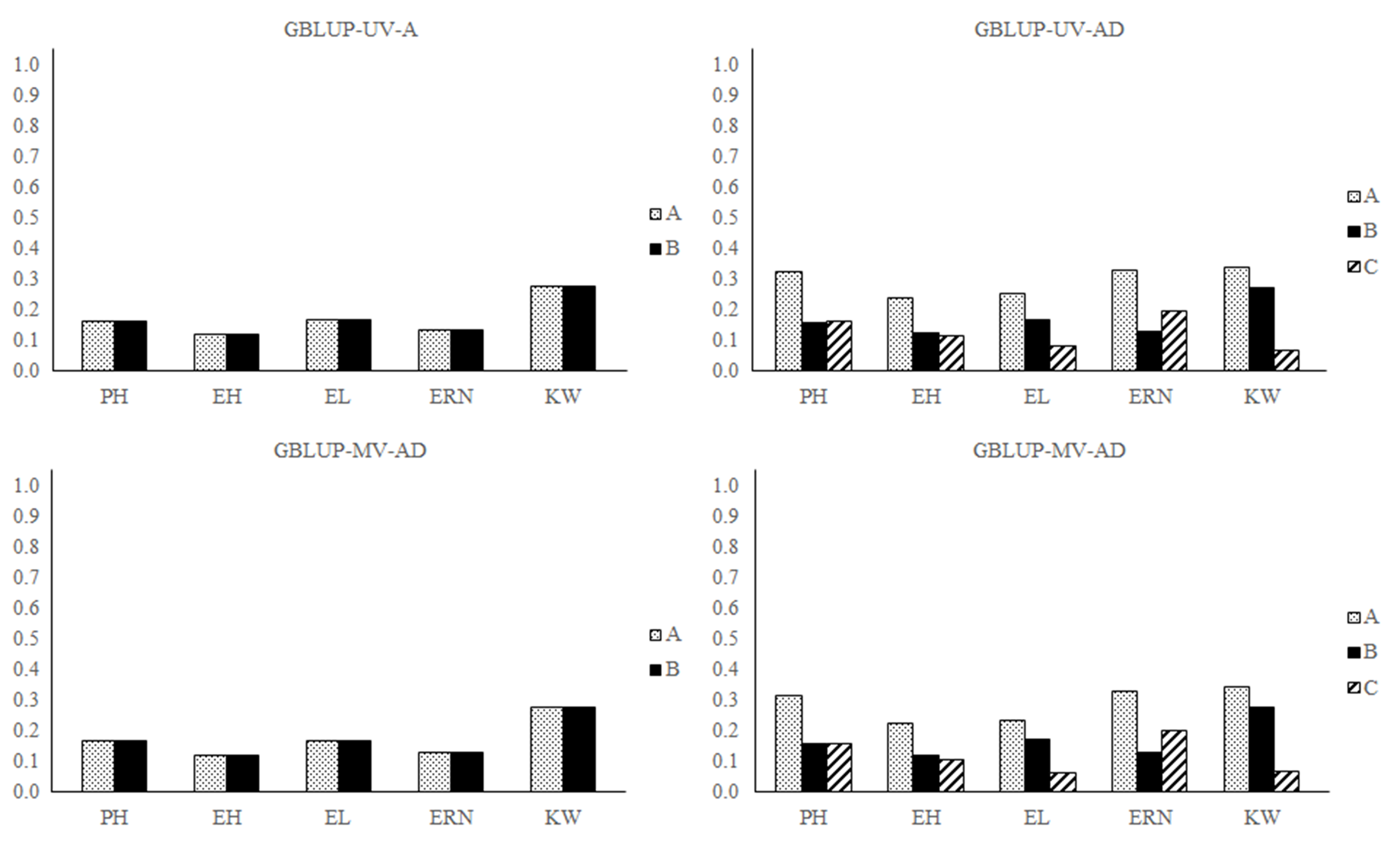

Supplement: S5 Fig — Models used: GBLUP-MV-A and GBLUP-MV-AD. Traits analyzed: Plant height (PH), ear height (EH), ear length (EL), ear row number (ERN) and kernel weight (KW). (A) Broad-sense heritability coefficients; (B) Additive heritability coefficients; (C) Dominance heritability coefficients. (TIF) [file pone.0152045.s005.tif]

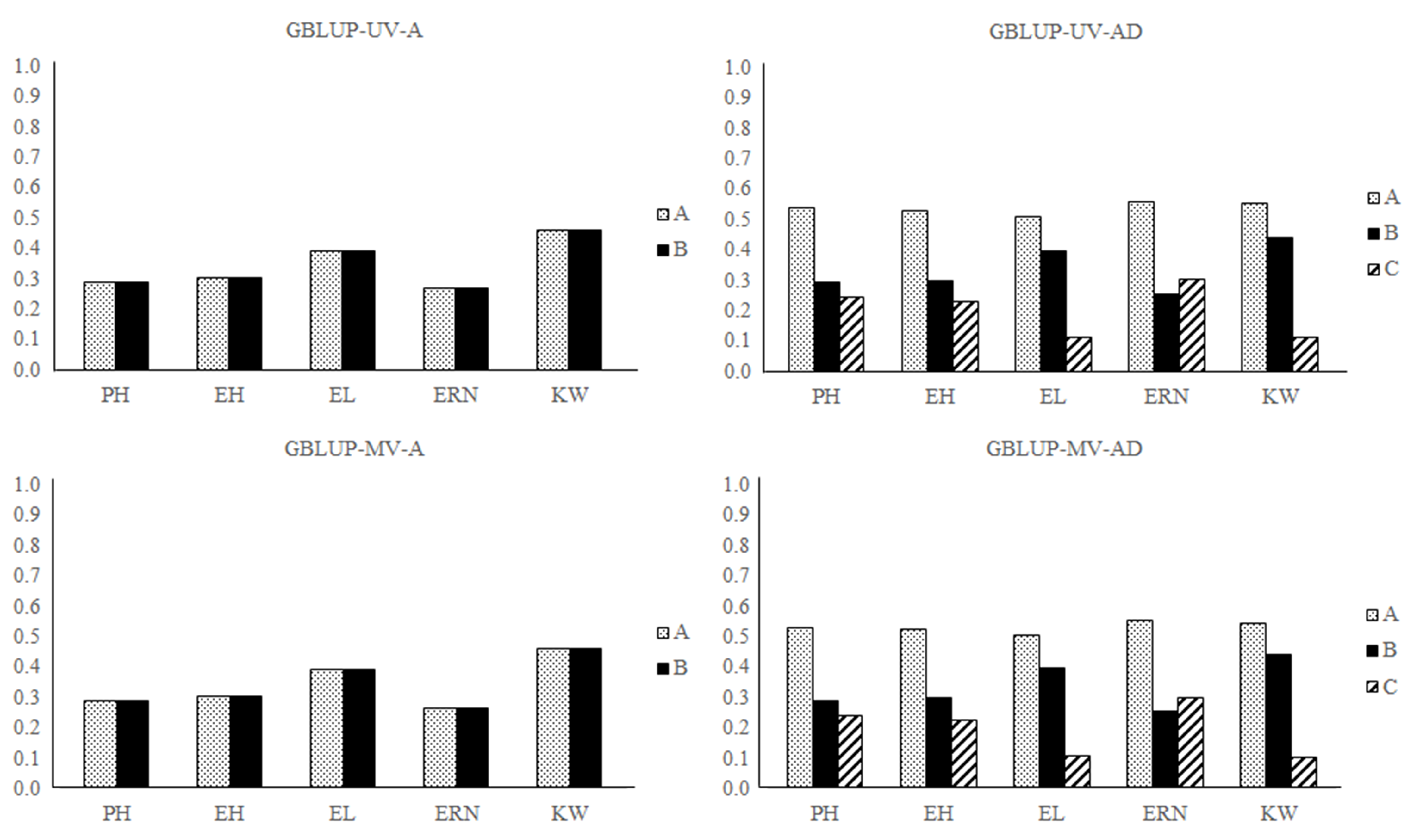

Supplement: S6 Fig — Models used: GBLUP-UV-A, GBLUP-UV-AD, GBLUP-MV-A and GBLUP-MV-AD. Traits analyzed: Plant height (PH), ear height (EH), ear length (EL), ear row number (ERN) and kernel weight (KW). (A) Broad-sense heritability coefficients; (B) Additive heritability coefficients; (C) Dominance heritability coefficients. (TIF) [file pone.0152045.s006.tif]

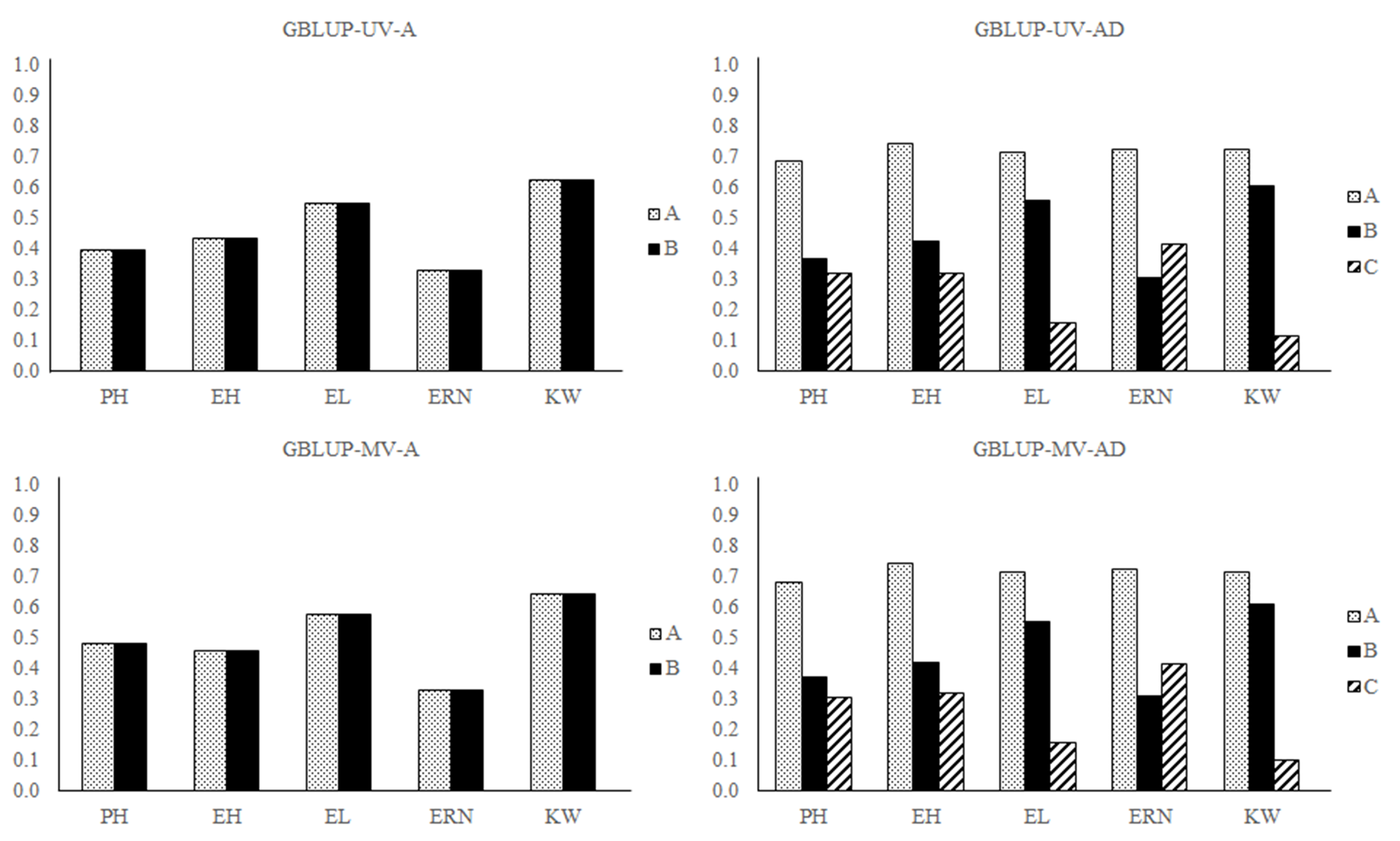

Supplement: S7 Fig — Models used: GBLUP-UV-A, GBLUP-UV-AD, GBLUP-MV-A and GBLUP-MV-AD. Traits analyzed: Plant height (PH), ear height (EH), ear length (EL), ear row number (ERN) and kernel weight (KW). (A) Broad-sense heritability coefficients; (B) Additive heritability coefficients; (C) Dominance heritability coefficients. (TIF) [file pone.0152045.s007.tif]

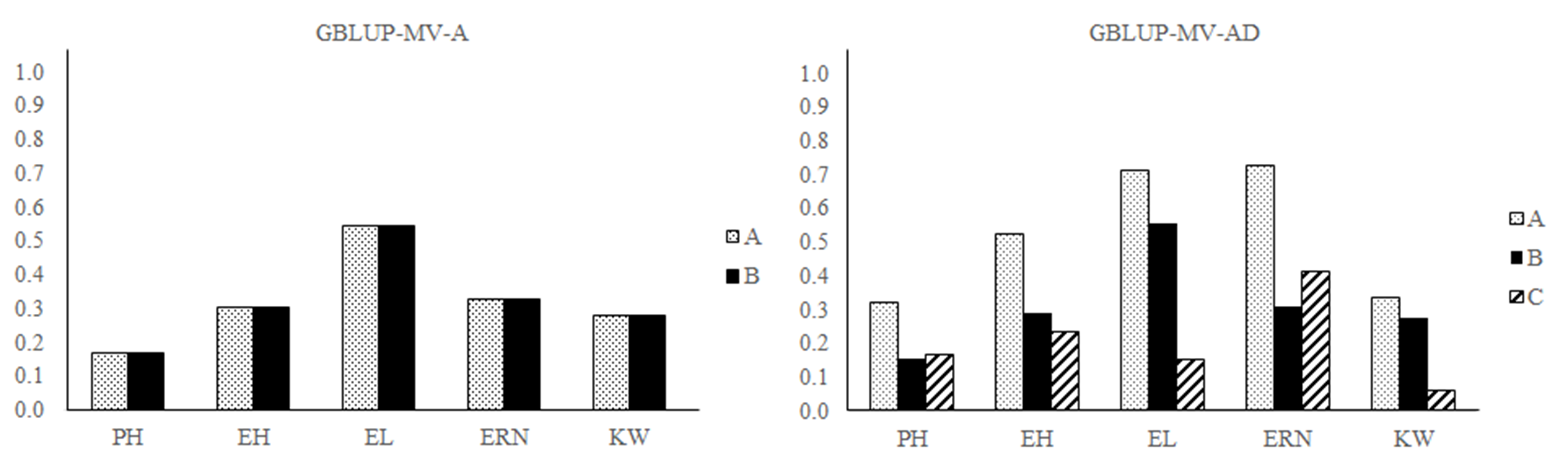

Supplement: S8 Fig — Models used: GBLUP-MV-A e GBLUP-MV-AD. Traits analyzed: Plant height (PH), ear height (EH), ear length (EL), ear row number (ERN) and kernel weight (KW). (A) Broad-sense heritability coefficients; (B) Additive heritability coefficients; (C) Dominance heritability coefficients. (TIF) [file pone.0152045.s008.tif]
