# Supplementary material for: Inclusion of Dominance Effects in the Multivariate GBLUP Model
Source: PLoS One. 2016 Apr 13;11(4):e0152045. doi: 10.1371/journal.pone.0152045 (PMC4830534; doi:10.1371/journal.pone.0152045)
Supplement: S1 Table — Traits: plant height (PH), ear height (EH), ear length (EL), ear row number (ERN), kernel weight (KW). (DOCX) [file pone.0152045.s009.docx]

**S1 Table. Additive correlations estimated between the five traits using GBLUP-MV-A.**

|  | **EH (0.3)** | **EL (0.3)** | **ERN (0.3)** | **KW (0.3)** |
| --- | --- | --- | --- | --- |
| **PH** | 0.6509 | -0.1717 | 0.0724 | 0.4664 |
| **EH** | - | -0.1570 | 0.6473 | 0.3153 |
| **EL** | - | - | -0.1639 | 0.3221 |
| **ERN** | - | - | - | -0.0555 |
|  | **EH (0.3)** | **EL (0.3)** | **ERN (0.3)** | **KW (0.3)** |
| **PH** | 0.6960 | -0.0135 | 0.1217 | 0.6115 |
| **EH** | - | 0.1260 | 0.3885 | 0.5712 |
| **EL** | - | - | -0.1458 | 0.3277 |
| **ERN** | - | - | - | 0.1334 |
|  | **EH (0.7)** | **EL (0.7)** | **ERN (0.7)** | **KW (0.7)** |
| **PH** | 0.9697 | 0.8405 | -0.5736 | 0.9245 |
| **EH** | - | 0.8433 | -0.4662 | 0.9009 |
| **EL** | - | - | -0.5699 | 0.8984 |
| **ERN** | - | - | - | -0.5550 |
|  | **EH (0.3)** | **EL (0.7)** | **ERN (0.7)** | **KW (0.3)** |
| **PH** | 0.6597 | -0.0403 | 0.0169 | 0.4772 |
| **EH** | - | 0.1147 | 0.5313 | 0.4913 |
| **EL** | - | - | -0.0590 | 0.3575 |
| **ERN** | - | - | - | 0.1020 |
|  | **EH (0.3)** | **EL (0.3)** | **ERN (0.3)** | **KW (0.3)** |
| **PH** | 0.6509 | -0.1717 | 0.0724 | 0.4664 |
| **EH** | - | -0.1570 | 0.6473 | 0.3153 |
| **EL** | - | - | -0.1639 | 0.3221 |
| **ERN** | - | - | - | -0.0555 |

Traits: plant height (PH), ear height (EH), ear length (EL), ear row number (ERN), kernel weight (KW)**.** The adjusted heritability values used during the data construction are in parentheses.
