# Supplementary material for: Inclusion of Dominance Effects in the Multivariate GBLUP Model
Source: PLoS One. 2016 Apr 13;11(4):e0152045. doi: 10.1371/journal.pone.0152045 (PMC4830534; doi:10.1371/journal.pone.0152045)
Supplement: S2 Table — Traits: plant height (PH), ear height (EH), ear length (EL), ear row number (ERN), kernel weight (KW). The adjusted heritability values used during the data construction are in parentheses. (DOCX) [file pone.0152045.s010.docx]

**S2 Table: Additive ($\alpha$), dominant (**$\delta$**) and total genetic (g) correlations estimated between the five traits using GBLUP-MV-AD.**

|  |  | **EH (0.3)** | **EL (0.3)** | **ERN (0.3)** | **KW (0.3)** |
| --- | --- | --- | --- | --- | --- |
| **PH** | $\alpha$ | 0.5928 | -0.1637 | 0.1143 | 0.4738 |
|  | $\delta$ | 0.5638 | 0.6535 | -0.3579 | 0.7463 |
|  | g | 0.5793 | 0.0768 | -0.0928 | 0.5336 |
| **EH** | $\alpha$ | - | -0.1634 | 0.6026 | 0.3065 |
|  | $\delta$ | - | 0.4079 | -0.4000 | 0.1723 |
|  | g | - | 0.0177 | 0.1379 | 0.2527 |
| **EL** | $\alpha$ | - | - | -0.1222 | 0.3480 |
|  | $\delta$ | - | - | 0.0697 | 0.8407 |
|  | g | - | - | -0.0591 | 0.4424 |
| **ERN** | $\alpha$ | - | - | - | -0.0945 |
|  | $\delta$ | - | - | - | 0.1492 |
|  | g | - | - | - | -0.0192 |
|  |  | **EH (0.5)** | **EL (0.5)** | **ERN (0.5)** | **KW (0.5)** |
| **PH** | $\alpha$ | 0.6447 | -0.0315 | 0.2026 | 0.5806 |
|  | $\delta$ | 0.4945 | 0.2024 | -0.2330 | -0.2486 |
|  | g | 0.5830 | 0.0227 | 0.0412 | 0.3770 |
| **EH** | $\alpha$ | - | 0.1418 | 0.4830 | 0.5512 |
|  | $\delta$ | - | 0.3236 | 0.0865 | -0.2716 |
|  | g | - | 0.1794 | 0.3204 | 0.3186 |
| **EL** | $\alpha$ | - | - | -0.1088 | 0.2999 |
|  | $\delta$ | - | - | 0.0208 | 0.6263 |
|  | g | - | - | -0.0744 | 0.3452 |
| **ERN** | $\alpha$ | - | - | - | 0.0950 |
|  | $\delta$ | - | - | - | 0.4852 |
|  | g | - | - | - | 0.1795 |
|  |  | **EH (0.7)** | **EL (0.7)** | **ERN (0.7)** | **KW (0.7)** |
| **PH** | $\alpha$ | 0.7374 | -0.1421 | 0.1467 | 0.3688 |
|  | $\delta$ | 0.6359 | 0.4804 | 0.1313 | 0.2385 |
|  | g | 0.6981 | 0.0051 | 0.1409 | 0.3265 |
| **EH** | $\alpha$ | - | 0.1246 | 0.3747 | 0.3355 |
|  | $\delta$ | - | 0.2371 | 0.1739 | -0.0574 |
|  | g | - | 0.1470 | 0.2954 | 0.2329 |
| **EL** | $\alpha$ | - | - | -0.1128 | 0.3831 |
|  | $\delta$ | - | - | 0.0963 | 0.6638 |
|  | g | - | - | -0.0584 | 0.4177 |
| **ERN** | $\alpha$ | - | - | - | -0.0036 |
|  | $\delta$ | - | - | - | 0.3197 |
|  | g | - | - | - | 0.0621 |
|  |  | **EH (0.3)** | **EL (0.7)** | **ERN (0.7)** | **KW (0.3)** |
| **PH** | $\alpha$ | 0.6162 | -0.0749 | 0.0958 | 0.4865 |
|  | $\delta$ | 0.5653 | 0.2262 | -0.2504 | 0.7575 |
|  | g | 0.5929 | 0.0072 | -0.0488 | 0.5444 |
| **EH** | $\alpha$ | - | 0.0757 | 0.5651 | 0.4894 |
|  | $\delta$ | - | 0.2178 | 0.0505 | 0.0969 |
|  | g | - | 0.1087 | 0.3440 | 0.3514 |
| **EL** | $\alpha$ | - | - | -0.1107 | 0.2918 |
|  | $\delta$ | - | - | 0.1175 | 0.4929 |
|  | g | - | - | -0.0511 | 0.3250 |
| **ERN** | $\alpha$ | - | - | - | 0.0837 |
|  | $\delta$ | - | - | - | -0.0480 |
|  | g | - | - | - | 0.0461 |

Traits: plant height (PH), ear height (EH), ear length (EL), ear row number (ERN), kernel weight (KW)**.** The adjusted heritability values used during the data construction are in parentheses.
