# Supplementary material for: Inclusion of Dominance Effects in the Multivariate GBLUP Model
Source: PLoS One. 2016 Apr 13;11(4):e0152045. doi: 10.1371/journal.pone.0152045 (PMC4830534; doi:10.1371/journal.pone.0152045)
Supplement: S3 Table — Traits: plant height (PH), ear height (EH), ear length (EL), ear row number (ERN), kernel weight (KW). The adjusted heritability values used during the data construction are in parentheses. (DOCX) [file pone.0152045.s011.docx]

**S3 Table. Additive, dominant and total genetic variance components obtained by the analysis of the inbred lines. These values were considered as true in single-cross hybrids analysis.**

|  | **PH** | **EH** | **EL** | **ERN** | **KW** |
| --- | --- | --- | --- | --- | --- |
| $\sigma_{\alpha}^{2}$ | 23.36 | 74.65 | 112.10 | 0.80 | 0.35 |
| $\sigma_{\delta}^{2}$ | 19.07 | 60.55 | 33.00 | 1.00 | 0.06 |
| $\sigma_{g}^{2}$ | 42.44 | 135.20 | 145.10 | 1.80 | 0.41 |

Traits: plant height (PH), ear height (EH), ear length (EL), ear row number (ERN), kernel weight (KW)**.**
