# Supplementary material for: Inclusion of Dominance Effects in the Multivariate GBLUP Model
Source: PLoS One. 2016 Apr 13;11(4):e0152045. doi: 10.1371/journal.pone.0152045 (PMC4830534; doi:10.1371/journal.pone.0152045)
Supplement: S5 Table — Estimated additive (σα2), dominance (σδ2) and total (σg2) genetic variance and sum of squares of additive (PRESSα), dominance (PRESSδ) and total genetic (PRESSg) predicted residual errors for the traits plant height (PH), ear height (EH), ear length (EL), ear row number (ERN), and kernel weight (KW). The adjusted heritability values used during the data construction are in parentheses. (DOCX) [file pone.0152045.s013.docx]

**S5 Table. Estimated and parametric genetic variance components and the sum of squares of the predicted error of genetic effects estimated using the GBLUP-MV-A and GBLUP-MV-AD.**

|  |  | **PH (0.3)** | **EH (0.3)** | **EL (0.3)** | **ERN (0.3)** | **KW (0.3)** |
| --- | --- | --- | --- | --- | --- | --- |
| **MV-A** | $\sigma_{\alpha}^{2}$ | 32.66 | 65.74 | 100.77 | 1.01 | 0.38 |
|  | $PRESS_{\alpha}$ | 1,552.80 | 6,880.80 | 8,538.30 | 32.00 | 13.50 |
|  | $PRESS_{g}$ | 7,944.50 | 29,703.10 | 21,293.90 | 415.60 | 36.10 |
| **MV-AD** | $\sigma_{\alpha}^{2}$ | 28.68 | 61.63 | 106.92 | 1.03 | 0.36 |
|  | $\sigma_{\delta}^{2}$ | 20.54 | 53.86 | 26.68 | 0.88 | 0.08 |
|  | $\sigma_{g}^{2}$ | 49.22 | 115.49 | 133.59 | 1.92 | 0.44 |
|  | $PRESS_{\alpha}$ | 1,627.20 | 5,617.29 | 8,605.41 | 34.93 | 14.14 |
|  | $PRESS_{\delta}$ | 4,769.87 | 12,022.08 | 6,336.99 | 119.64 | 16.12 |
|  | $PRESS_{g}$ | 5,105.89 | 17,095.04 | 14,013.16 | 132.14 | 27.45 |
|  |  | PH (0.3) | EH (0.3) | EL (0.3) | ERN (0.3) | KW (0.3) |
| **MV-A** | $\sigma_{\alpha}^{2}$ | 48.22 | 101.76 | 138.12 | 1.24 | 0.44 |
|  | $PRESS_{\alpha}$ | 1,013.60 | 2,481.10 | 2,495.70 | 13.80 | 6.90 |
|  | $PRESS_{g}$ | 7,189.60 | 25,108.60 | 14,801.90 | 393.40 | 26.90 |
| **MV-AD** | $\sigma_{\alpha}^{2}$ | 40.57 | 86.30 | 138.32 | 1.16 | 0.36 |
|  | $\sigma_{\delta}^{2}$ | 22.89 | 67.06 | 23.09 | 0.71 | 0.06 |
|  | $\sigma_{g}^{2}$ | 63.46 | 153.36 | 161.40 | 1.87 | 0.42 |
|  | $PRESS_{\alpha}$ | 872.18 | 2,001.84 | 2,516.43 | 16.66 | 5.60 |
|  | $PRESS_{\delta}$ | 2,272.55 | 7,112.43 | 4,867.94 | 82.66 | 11.56 |
|  | $PRESS_{g}$ | 2,387.58 | 8,386.19 | 6,712.12 | 89.22 | 16.19 |
|  |  | PH (0.7) | EH (0.7) | EL (0.7) | ERN (0.7) | KW (0.7) |
| **MV-A** | $\sigma_{\alpha}^{2}$ | 569.03 | 737.22 | 766.66 | 1.70 | 2.59 |
|  | $PRESS_{\alpha}$ | 2,158.40 | 2,160.30 | 1,908.60 | 11.70 | 6.60 |
|  | $PRESS_{g}$ | 5,229.30 | 22,562.40 | 11,294.90 | 386.10 | 21.90 |
| **MV-AD** | $\sigma_{\alpha}^{2}$ | 33.52 | 86.84 | 145.80 | 0.89 | 0.36 |
|  | $\sigma_{\delta}^{2}$ | 18.38 | 58.87 | 25.56 | 0.56 | 0.04 |
|  | $\sigma_{g}^{2}$ | 51.90 | 145.70 | 171.36 | 1.46 | 0.40 |
|  | $PRESS_{\alpha}$ | 738.25 | 881.22 | 821.72 | 10.10 | 3.24 |
|  | $PRESS_{\delta}$ | 1,792.12 | 5,071.81 | 3,428.82 | 47.17 | 5.63 |
|  | $PRESS_{g}$ | 1,758.59 | 5,356.71 | 3,713.70 | 47.33 | 7.75 |
|  |  | PH (0.3) | EH (0.3) | EL (0.7) | ERN (0.7) | KW (0.3) |
| **MV-A** | $\sigma_{\alpha}^{2}$ | 32.54 | 93.66 | 172.15 | 1.05 | 0.39 |
|  | $PRESS_{\alpha}$ | 1,811.50 | 2,752.40 | 841.40 | 12.40 | 13.80 |
|  | $PRESS_{g}$ | 8,150.80 | 25,369.80 | 12,799.70 | 388.50 | 35.60 |
| **MV-AD** | $\sigma_{\alpha}^{2}$ | 27.70 | 78.38 | 148.17 | 0.91 | 0.35 |
|  | $\sigma_{\delta}^{2}$ | 22.27 | 68.27 | 26.27 | 0.58 | 0.08 |
|  | $\sigma_{g}^{2}$ | 49.97 | 146.65 | 174.45 | 1.49 | 0.43 |
|  | $PRESS_{\alpha}$ | 1,739.31 | 2360.33 | 857.16 | 10.07 | 13.43 |
|  | $PRESS_{\delta}$ | 4,559.40 | 7,967.44 | 3,106.47 | 45.96 | 15.25 |
|  | $PRESS_{g}$ | 5,096.80 | 9,275.91 | 3,411.84 | 47.33 | 26.52 |

Estimated additive ($\sigma_{\alpha}^{2}$ ), dominance ($\sigma_{\delta}^{2}$ ) and total ($\sigma_{g}^{2}$ ) genetic variance and sum of squares of additive ($PRESS_{\alpha}$ ), dominance ($PRESS_{\delta}$) and total genetic ($PRESS_{g}$) predicted residual errors for the traits plant height (PH), ear height (EH), ear length (EL), ear row number (ERN), and kernel weight (KW)**.** The adjusted heritability values used during the data construction are in parentheses.
