# Supplementary material for: Inclusion of Dominance Effects in the Multivariate GBLUP Model
Source: PLoS One. 2016 Apr 13;11(4):e0152045. doi: 10.1371/journal.pone.0152045 (PMC4830534; doi:10.1371/journal.pone.0152045)
Supplement: S1 Text — (DOCX) [file pone.0152045.s014.docx]

**Text S1. Weighting additive and dominant values for the markers**

The weighted additive effect considering the 100 selected markers obtained using Bayes B method was given by:

, where $h_{A}^{2}=\frac{\sum_{i=1}^{100} \sigma_{a}^{2}+\sum_{i=1}^{100} \sigma_{d}^{2}}{\sum_{i=1}^{100} \sigma_{a}^{2}+\sum_{i=1}^{100} \sigma_{d}^{2}+\sigma_{e}^{2}}$ and

Where $a_{rk}$ is the reparametrized allelic substitution related to the k-th marker, $h_{A}^{2}$ is the broad-sense heritability for all selected makers, is the broad-sense heritability considering the remain 99 markers out $a_{rk}$, $\sum_{i=1}^{100} \sigma_{a}^{2}$and $\sum_{i=1}^{100} \sigma_{d}^{2}$are the total additive and dominant variances and $\sum_{i=1}^{99} \sigma_{a\neq k}^{2}$ is total additive variance out $\sigma_{a_{rk}}^{2}$ and $\sigma_{e}^{2}$ os the residual variance estimated by Bayes B method.

Same approach was applied to dominance effects. That is, the weighted dominance effect was given by:

, em que $h_{A}^{2}=\frac{\sum_{i=1}^{100} \sigma_{a}^{2}+\sum_{i=1}^{100} \sigma_{d}^{2}}{\sum_{i=1}^{100} \sigma_{a}^{2}+\sum_{i=1}^{100} \sigma_{d}^{2}+\sigma_{e}^{2}}$ e

Where $d_{rk}$ is the weighted dominance effect related to k-th marker; is the broad-sense heritability out of $d_{k}$ and $\sum_{i=1}^{99} \sigma_{d\neq k}^{2}$ is the dominance variance out of $\sigma_{dk}^{2}$.
